# Click Chemistry Mediated Immune Synapse Augmentation in Natural Killer Cell–Cancer Membrane Engagement and Facilitated Anticancer Efficacies of Natural Killer Cell Therapy

**DOI:** 10.34133/bmr.0376

**Published:** 2026-06-09

**Authors:** Kyung Mu Noh, Ashok Kumar Jangid, Eunha Kim, Kyobum Kim

**Affiliations:** ^1^ Department of Chemical and Biochemical Engineering, Dongguk University, Seoul, Republic of Korea.; ^2^ Cellbastian Inc., Seoul, Republic of Korea.

## Abstract

Non-small cell lung cancer remains a major clinical challenge due to aggressive metastasis and limited therapeutic modalities. The innate cytotoxicity of natural killer (NK) cells and their ability to eliminate malignant cells in an antigen-independent manner have attracted considerable interest for cancer immunotherapy. However, the therapeutic performance of NK cells in solid tumors is severely constrained by (a) tumor heterogeneity, (b) physical barriers within the tumor microenvironment, and (c) insufficient tumor-targeting specificity. To address these challenges, we here develop a lipid biomaterial that enables the simultaneous surface engineering of cancer and NK cells to enhance their physical engagement within complex tumor microenvironments. The developed lipid biomaterials are composed of (a) a lipid moiety for stable surface anchoring and (2) complementary dibenzocyclooctyne (DBCO) and azide (N_3_) functional groups to mediate bioorthogonal click-reaction-driven cell–cell interactions. This modular design allows the rapid and non-genetic engineering of cell surfaces, promoting tumor-specific targeting through DBCO–N_3_ click reactions, while simultaneously enhancing NK cell activation and cytotoxic function. Surface engineering of non-small cell lung cancer cells (N_3_-cancer) and NK cells (D-NK) using lipid-N_3_ and lipid-DBCO substantially improved cancer recognition, immune activation, and NK cell-mediated cytotoxicity. Moreover, lipid-N_3_ successfully labeled 3-dimensional lung tumoroids embedded in collagen hydrogels that recapitulate the structure of native lung tissue, leading to superior antitumor efficacy upon interaction with D-NK cells. Collectively, this lipid-biomaterial-based strategy provides a versatile, receptor-independent approach to augment NK cell-based immunotherapy against heterogeneous solid tumors, offering a promising approach that avoids reliance on predefined tumor-specific ligand–receptor pairs.

## Introduction

Non-small cell lung cancer (NSCLC) accounts for approximately 85% of all lung cancer cases and remains a leading cause of cancer-related mortality worldwide [1]. Despite advances in targeted therapy and immune checkpoint blockade, the overall prognosis for patients with advanced NSCLC remains poor, largely due to tumor heterogeneity, the immunosuppressive tumor microenvironment (TME), and insufficient response durability [2]. These limitations highlight the urgent need for innovative strategies that can reprogram immune–tumor interactions, particularly via localized infiltration into the solid tumor region of NSCLC [3].

Adoptive cell therapy, including natural killer (NK) cell-based immunotherapy, has emerged as a promising modality to treat solid tumors. NK cells possess intrinsic cytotoxicity, do not require prior antigen priming, and exhibit low risk of off-target effects compared to T cell-based approaches [4,5]. However, the clinical outcomes of NK-cell therapy against NSCLC remain constrained. Major barriers include limited NK cell infiltration into tumor sites, weak immunological synapse (IS) formation, and the insufficient expression of tumor-retargeting ligands on NK cell surfaces, which collectively result in inadequate tumor recognition and reduced cytotoxic activity [6].

Notably, most current NK cell engineering strategies rely on the unidirectional modification of immune effector cells, aiming to enhance target recognition ability [7–9]. These approaches have demonstrated promising therapeutic potential. However, in heterogeneous solid tumors, cancer cell populations may exhibit variable expression of targetable surface markers [10], which can potentially limit the uniform effectiveness of such strategies. As these approaches rely on predefined tumor-associated markers, antigen-negative or low-expression tumor subpopulations may partially evade immune recognition, thereby reducing overall targeting efficiency and therapeutic outcomes [11]. These limitations suggest that, in addition to immune cell-centric engineering approaches, complementary strategies that modulate both immune effector cells and tumor cells may further enhance cell–cell interactions at the interface. In this context, simultaneous and coordinated surface engineering of both NK cells and cancer cells, independent of specific biomarker expression, may provide an alternative strategy to improve targeting efficiency in heterogeneous TMEs.

To address these challenges, recent advances in cell surface engineering to enhance effector-to-target cellular interactions have introduced strategies like bispecific engagers [12], nanobridge-forming nanoparticles [13], chimeric antigen receptor engineering [14], and bioorthogonal click chemistry to artificially strengthen cell–cell interactions [15]. Although to some extent these approaches improve target specificity, they often require complex bio-conjugation steps, genetic modification, and unpredictable metabolic manipulation [16,17]. In contrast, amphiphilic lipid-mediated membrane anchoring provides sufficient ligand presentation onto cell surfaces for IS engagement via bioorthogonality. In this study, we develop the rapid, universal, and non-genetic surface engineering of both NK cells and cancer cells via hydrophobic interaction-driven membrane anchoring. Dibenzocyclooctyne (DBCO)–azide (N_3_) click chemistry confers several distinct advantages: (a) the reaction proceeds rapidly under physiological conditions (rate constants of 1 to 60 M^−1^·s^−1^), without interfering with native biochemical processes or requiring cytotoxic copper catalysts [18]; (b) DBCO–N_3_ reactions form covalent bonds with superior stability and retention, compared to the transient binding characteristic of endogenous ligand–receptor interactions [19]; and (c) DBCO and N_3_ functional groups can be stably displayed on cell membranes, enabling highly selective, proximity-driven conjugation at cell–cell interfaces [20]. Therefore, the integration of DBCO–N_3_ click chemistry with lipid-mediated cell surface engineering platform allows the facile incorporation of reactive functional groups (i.e., DBCO and N_3_) onto living cell surfaces without genetic manipulation, provides exceptional specificity and stability, and markedly enhances NK–cancer cell physical engagement in a receptor/ligand-independent manner, thereby offering a powerful and broadly applicable strategy for cancer immunotherapy.

**Fig. 1.**
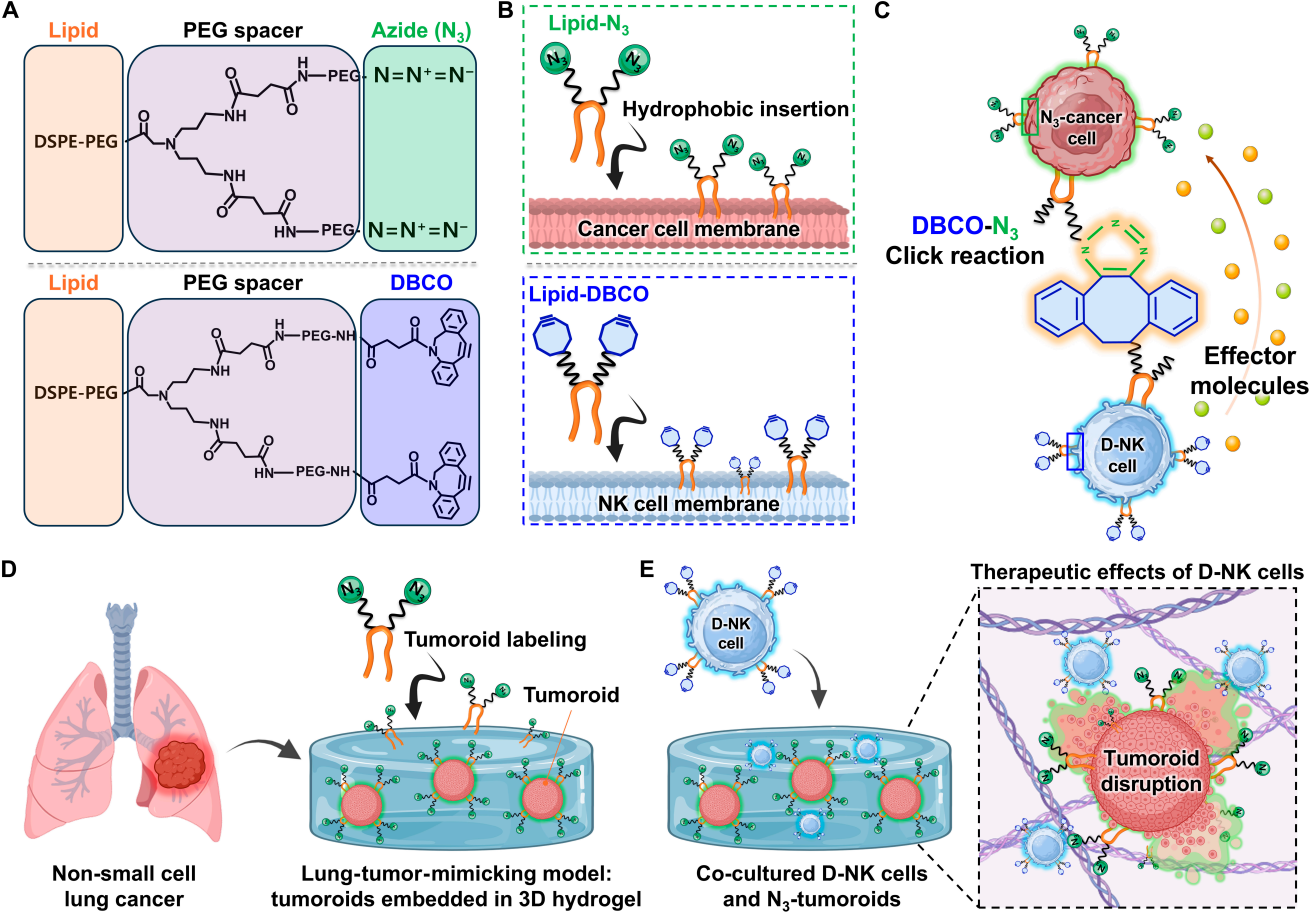
Lipid-biomaterial-mediated membrane engineering of cancer and natural killer (NK) cells, respectively, and dibenzocyclooctyne (DBCO)-azide (N_3_) click reaction-driven enhancement of antitumor efficacy against non-small cell lung cancer (NSCLC). (A) Chemical structures of lipid-N_3_ and lipid-DBCO biomaterials used to surface engineer cancer cells and NK cells, respectively. PEG, polyethylene glycol. (B) Schematic of the rapid, non-genetic membrane engineering of cancer cells and NK cells via the hydrophobic insertion of lipid biomaterials into the plasma membrane. (C) Reinforcement of immunological synapse (IS) formation through DBCO-N_3_ click reactions between lipid-N_3_-coated cancer cells (N_3_-cancer) and lipid-DBCO-engineered NK cells (D-NK), leading to stabilized cell–cell physical engagement. (D) Biomimetic 3-dimensional (3D) lung tumor model consisting of tumoroids embedded within a type I collagen hydrogel and surface labeling of tumoroids with lipid-N_3_ within the complex collagen matrix. (E) Enhanced antitumor activity of D-NK cells against lipid-N_3_-labeled tumoroids in a 3D collagen hydrogel, resulting from reinforced physical engagement and augmented IS formation.

In this study, we applied DBCO–N_3_ click chemistry to enhance NK–cancer cell interactions at the membrane interface (Fig. 1). NK cells were engineered with lipid-DBCO (D-NK cells), while NSCLC cells were coated with lipid-N_3_ (N_3_-cancer cells) (Fig. 1A and B), enabling selective and efficient DBCO–N_3_ reaction upon cell–cell contact. This lipid-mediated bidirectional surface engineering significantly (a) strengthened IS formation at NK–tumor interfaces, (b) enhanced the activation of NK cells and increased the secretion of cytotoxic cytokines and lytic granules, and (c) ultimately improved cancer cell killing efficiency (Fig. 1C). Furthermore, to evaluate the therapeutic potential under physiologically relevant conditions, we established a 3-dimensional (3D) collagen-based lung tumoroid model that recapitulates the elasticity and microarchitecture of native lung tissue (Fig. 1D) [21]. Importantly, the collagen-based 3D tumoroid model recapitulates both the mechanical stiffness and nanofibrous microarchitecture of native lung tissue and the associated extracellular matrix (ECM), which imposes physiologically relevant diffusion constraints. Accordingly, evaluating lipid-biomaterial transport, membrane insertion, and surface immobilization within this system enables assessment of their anchoring efficiency and bioorthogonal reactivity under conditions that closely approximate in vivo ECM-mediated physical barriers. Our findings demonstrate that the lipid-N_3_ biomaterials effectively penetrated and coated 3D tumoroids within the collagen matrix. Notably, D-NK cells exhibited robust binding to N_3_-tumoroids and achieved markedly enhanced cytotoxicity, even within this lung tissue mimicking 3D TME (Fig. 1E). Therefore, this work presents a bidirectional lipid–click chemistry platform that enables receptor-independent physical engagement between NK and cancer cells, thereby augmenting immune interactions and overcoming major barriers in NK cell immunotherapy for NSCLC.

## Materials and Methods

### Materials

1,2-Distearoyl-sn-glycero-3-phosphoethanolamine (DSPE)-polyethylene glycol amine 2000 (PEG2k-amine; LP096005-2k), DBCO-acid, azide-polyethylene glycol amine 2000 (azide-PEG2k-amine), and DBCO-amine were obtained from Biopharma PEG Scientific Inc. (USA). The 1-ethyl-3-(3-(dimethylamino)propyl) carbodiimide (EDC, E7750), *N*-hydroxysuccinimide (NHS), *N*,*N*-*bis*(*N*′-Fmoc-3-aminopropyl)glycine potassium hemisulfate (Di-Fmoc-Gly), anhydrous dimethylformamide (DMF), 4-dimethylaminopyridine (DMAP), *bis*-polyethylene glycol amine 2000 (bis-PEG2k-amine), and deuterated nuclear magnetic resonance (NMR) solvent were purchased from Sigma-Aldrich. Spectrum dialysis membrane tubing was purchased from Thermo Fisher Scientific.

### Synthesis of lipid-N_3_ and lipid-DBCO

**Fig. 2.**
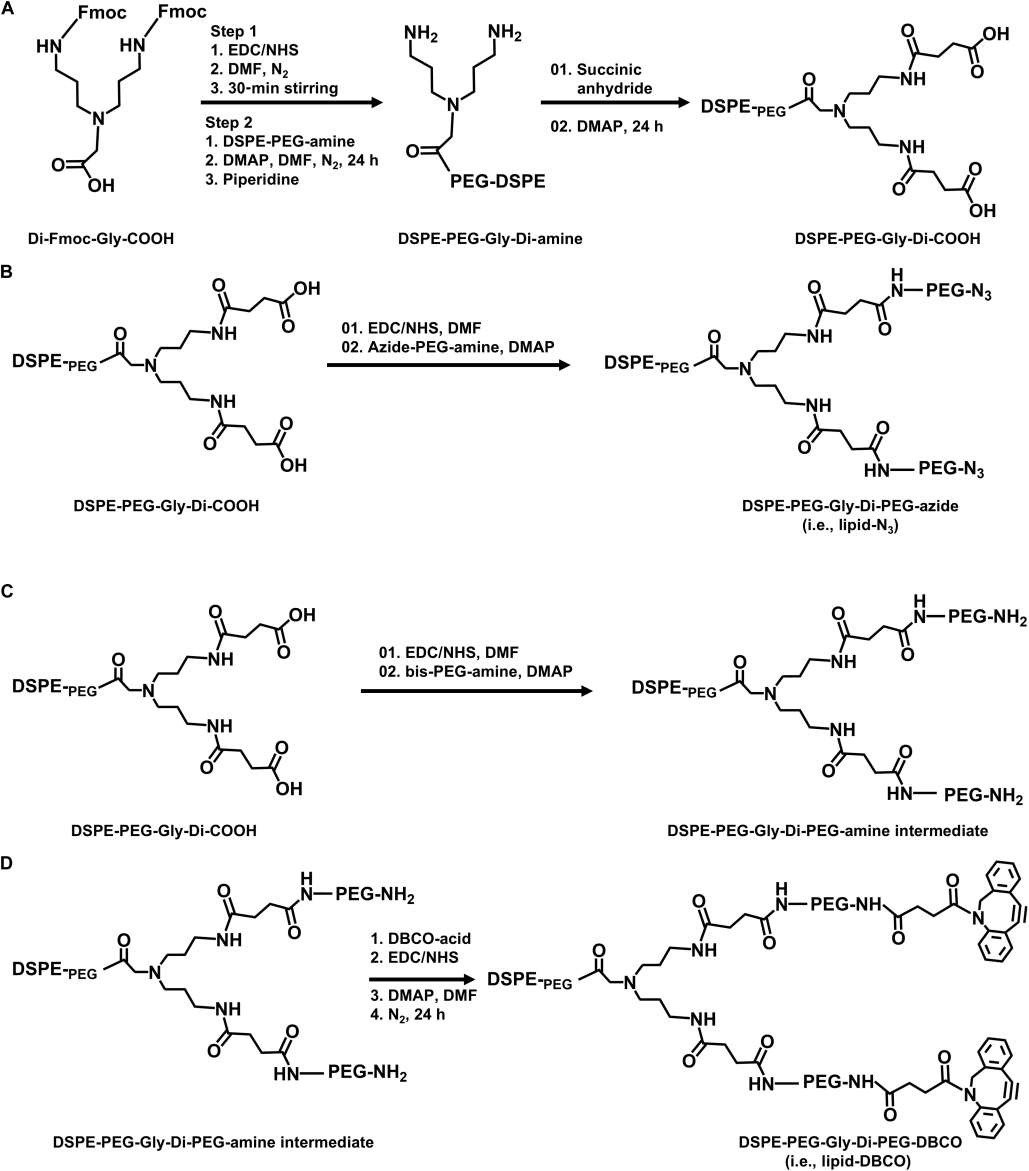
Synthetic route of the lipid-N_3_ and lipid-DBCO biomaterial for cancer cell and NK cell surface engineering, respectively. (A) DSPE-PEG-amine conversion into DSPE-PEG-Gly-Di-COOH via Di-Fmoc-Gly-COOH conjugation using 1-ethyl-3-(3-(dimethylamino)propyl) carbodiimide (EDC)/*N*-hydroxysuccinimide (NHS) coupling reaction, followed by Fmoc deprotection, and succinylation reaction, and (B) synthesis of the DSPE-PEG-Gly-Di-PEG-azide (lipid-N_3_) biomaterial via azide-PEG-amine conjugation with DSPE-PEG-Gly-Di-COOH using EDC/NHS coupling reaction. (C) Synthesis of the DSPE-PEG-Gly-Di-PEG-amine intermediate via bis-PEG-amine conjugation with DSPE-PEG-Gly-Di-COOH using EDC/NHS coupling reaction and (D) synthesis of DSPE-PEG-Gly-Di-PEG-DBCO (i.e., lipid-DBCO) biomaterial using DBCO-acid conjugation with DSPE-PEG-Gly-Di-PEG-amine intermediate via EDC/NHS coupling reaction. DMAP, 4-dimethylaminopyridine; DMF, dimethylformamide.

The lipid-N_3_ and lipid-DBCO biomaterials were synthesized as described in our previous report, with modifications [22,23] (Fig. 2). In brief, 124.2 mg (1.2 eq) of *N*,*N*-*bis*(*N*′-Fmoc-3-aminopropyl) glycine potassium hemisulfate (Di-Fmoc-Gly-COOH), 37.1 mg EDC (1.2 eq), and 46.4 mg (1.5 eq) NHS were dissolved in anhydrous DMF and allowed to react for 30 min under N_2_ protection. Subsequently, 300 mg of DSPE-PEG-amine was added, along with a catalytic amount of DMAP (5 mg). The reaction mixture was stirred at room temperature (RT) for 24 h under a N_2_ atmosphere. Piperidine (1 ml) was then added to deprotect the Fmoc group. The resulting DSPE-PEG-Gly-Di-amine was precipitated using cold diethyl ether 3 times and dialyzed (molecular weight cut-off [MWCO] 2 kDa) in deionized water (DW) for 1 d to remove impurities. Next, DSPE-PEG-Gly-Di-amine was converted to DSPE-PEG-Gly-Di-COOH by reaction with succinic anhydride in the presence of DMAP for 24 h under N_2_ protection (Fig. 2A). The reaction mixture was subsequently dialyzed (MWCO 2 kDa) in DW for 2 d for further purification, followed by lyophilization to yield a powdered product. Subsequently, 100 mg of DSPE-PEG-Gly-Di-COOH with excess amounts of 15 mg EDC and 12 mg NHS were dissolved in anhydrous DMF and reacted for 30 min under N_2_ protection. Following this, 140.1 mg (2.2 eq) of azide-PEG-amine was added, along with a catalytic quantity 5 mg of DMAP. The reaction mixture was stirred at RT for 24 h under N_2_. The mixture was then dialyzed (MWCO 3.5 kDa) in DW for 3 d to remove the unreacted impurities, after which it was lyophilized to obtain the solid powdered DSPE-PEG-Gly-Di-PEG-azide (i.e., lipid-N_3_) biomaterial (Fig. 2B).

For the lipid-DBCO biomaterial synthesis, DSPE-PEG-Gly-Di-COOH reacted with bis-PEG-amine and further reacted with DBCO-acid (Fig. 2C). The DSPE-PEG-Gly-Di-COOH (100 mg) with excess amounts of 15 mg EDC and 13 mg NHS were dissolved in anhydrous DMF and reacted for 30 min under N_2_ protection. Then, 140.1 mg (2.2 eq) of bis-PEG-amine was added, along with a catalytic quantity of 5 mg of DMAP. The reaction mixture was stirred at RT for 24 h under N_2_. For DBCO conjugation, 6.4 mg DBCO-acid, 15 mg EDC, and 13 mg NHS were dissolved in 1 ml of anhydrous DMF and allowed to react for 30 min at RT under N_2_. Thereafter, 50 mg of DSPE-PEG-Gly-Di-PEG-amine and 5 mg of DMAP were added, and the reaction mixture was stirred at RT for 24 h (Fig. 2D). This mixture was then dialyzed (MWCO 2 kDa) in DW for 2 d to remove unreacted impurities. The synthesized biomaterials DSPE-PEG-Gly-Di-PEG-azide (lipid-N_3_) and DSPE-PEG-Gly-Di-PEG-DBCO (lipid-DBCO) were characterized by ^1^H NMR (500 MHz FT-NMR spectrometer; Bruker) and Fourier-transform infrared spectroscopy (FTIR) (PerkinElmer FTIR Spectrum Two; PerkinElmer) analysis. The NMR spectra were plotted and analyzed using MestReNova 6.0.0-5475 software.

### Cell culture

To culture NK-92MI cells (ATCC), complete growth media was used, comprising Minimum Essential Medium Alpha (Gibco) supplemented with 12.5% fetal bovine serum (FBS; Gibco), 12.5% horse serum (Gibco), 1% penicillin–streptomycin (P/S) solution (Corning), 0.2 mM myo-inositol (Sigma-Aldrich), 0.1 mM β-mercaptoethanol (Sigma-Aldrich), and 0.02 mM folic acid (Sigma-Aldrich). A549 (Korean Cell Line Bank) and NCI-H460 (Korean Cell Line Bank) were cultured in RPMI 1640 medium (Gibco) supplemented with 10% FBS (Gibco) and 1% P/S solution. MRC-5 (Korean Cell Line Bank) was cultured in high-glucose Dulbecco’s modified Eagle’s medium (Corning), supplemented with 10% FBS (Corning) and 1% P/S solution. Human umbilical vein endothelial cells (HUVEC; Lonza) were cultured in endothelial growth medium (EGM) consisting of EGM-2 SingleQuots Bullet Kits (Lonza) and 1% P/S solution. All cells were maintained at 37 °C in a humidified atmosphere with 5% CO_2_.

### Surface engineering and characterization of cancer cells and NK cells

Surface engineering of NK cells and NSCLC cells was performed using lipid biomaterials (lipid-DBCO or lipid-N_3_) through a one-step membrane insertion. To determine the optimal coating concentration, 1 × 10^6^ cancer cells or NK cells were incubated with 200 μl of 5-carboxyfluorescein (CFL)-conjugated lipid biomaterial (lipid-CFL) solutions (0–1.0 mg/ml) at RT for 30 min, followed by 2 washes with serum-free media. Fluorescence intensities of coated cells were quantified using flow cytometry (Beckman Coulter). For visualization, cancer cells and NK cells coated with 0.75 mg/ml lipid-CFL for 30 min were imaged by fluorescence microscopy (Ti-E System; Nikon), and fluorescence signals were analyzed using ImageJ software. Based on mean fluorescence intensity (MFI) quantification, target cells or NK cells were coated with lipid-N_3_ or lipid-DBCO at 0.75 mg/ml, corresponding to 0.75 mg per 5 × 10^6^ cells for all subsequent experiments. To evaluate coating retention, cancer cells and NK cells coated with 0.75 mg/ml lipid-CFL were cultured at 37 °C, and the MFI was measured by flow cytometry at predetermined time points of 0, 12, 24, 48, and 72 h.

### Cell coating efficiency

The amount of lipid biomaterials coated onto cancer cells and NK cells was quantified using lipid-CFL. After coating 1 × 10^6^ cells with 0.75 mg/ml lipid-CFL as previously described, cells were lysed in 250 μl of radioimmunoprecipitation assay buffer (ELPIS-BIOTECH) for 30 min at 4 °C. The fluorescence intensity of the lysates was measured by SpectraMax iD3 (excitation/emission = 485/535 nm). To consider background interference, fluorescence intensity of uncoated cell lysates was acquired in parallel. The amount of membrane-inserted lipid-CFL was calculated using a standard curve generated from serial dilutions of lipid-CFL.

### Membrane-anchoring efficiency of lipid biomaterials under high-serum conditions

To evaluate the membrane-anchoring efficiency of lipid biomaterials under high-serum conditions, A549 cancer cells were incubated in media containing 0% to 50% FBS to mimic high-serum environments. The cells were subsequently treated with lipid-CFL at a final concentration of 0.75 mg/ml and incubated for 30 min at RT. After incubation, the cells were washed with serum-free medium twice. The membrane-associated fluorescence intensity of lipid-CFL was then quantified by flow cytometry to determine the efficiency of lipid biomaterial anchoring on the cancer cell surface.

### Viability and proliferation capacity of the lipid-N_3_-coated cancer cells and lipid-DBCO-coated NK cells

Cancer cells coated with lipid-N_3_ (i.e., N_3_-A549 or N_3_-H460) and NK cells coated with lipid-DBCO (i.e., D-NK) were prepared as previously described. For cancer cells, 1 × 10^4^ N_3_-A549 or N_3_-H460 cells were seeded into flat-bottom 96-well plates and incubated in growth media at 37 °C for 24 h. For NK cells, 5 × 10^4^ D-NK cells were seeded into U-bottom 96-well plates and cultured in complete NK growth medium at 37 °C for 48 h. Cell viability and proliferation were assessed using EZ-Cytox WST-1 assay (DoGenBio), and absorbance values were normalized to 0 h readings. In addition, live/dead assay was performed for lipid-N_3_-coated cancer cells. Briefly, non-coated and lipid-N_3_-coated cancer cells were incubated with 2 μM of Calcein-AM and 4 μM of ethidium homodimer-1 solution in serum-free medium at RT for 30 min, washed twice, and imaged by fluorescence microscopy.

### Surface ligand/receptor availability

To evaluate whether lipid biomaterials (lipid-N_3_ or lipid-DBCO) affect membrane ligand/receptor availability, cancer cell receptors (death receptor 5 [DR5], Fas, and MICA/B) and NK cell ligands (tumor necrosis factor-related apoptosis-inducing ligand [TRAIL] and Fas ligand [FasL]) and activation marker (CD69) were analyzed by flow cytometry. Non-coated cells and lipid-N_3_ or lipid-DBCO-coated cells were incubated with PE-anti-human DR5 (Invitrogen), APC-anti-human CD95 (Fas) (Invitrogen), and APC-anti-human MICA/MICB antibodies (BioLegend) or APC-anti-human TRAIL (BD Biosciences), APC-anti-human FasL (BD Biosciences), and APC-anti-human CD69 antibodies (BioLegend) at 4 °C for 30 min. After 2 washes with cold fluorescence-activated cell sorting buffer (Dulbecco’s phosphate-buffered saline containing 2% FBS), cells were analyzed by flow cytometry to determine the expression and accessibility of cancer cell and NK cell surface ligands/receptors following lipid-biomaterial coating. In addition, to evaluate nonspecific activation of NK cells following lipid-DBCO coating, NK and D-NK cells were cultured in NK cell complete growth media for 48 h, and then surface expression levels of FasL, TRAIL, and CD69 were analyzed through flow cytometry.

### NK cell migration assay

Transwell migration assay was performed using transwell inserts (SPL, 35824) with an 8-μm pore size fully coated with 50% Matrigel (Corning, 254230) to mimic the physical barrier of the TME. NK or D-NK cells (2.5 × 10^5^ cells/insert well) were seeded in the upper chamber, while the bottom chamber contained NK cell complete growth medium supplemented with 100 ng/ml of CXCL10 as a chemoattractant [24]. After 24 h of incubation, migrated cells were collected from the lower chamber and counted manually.

### Bioorthogonal reactivity at the cell membrane interfaces

The bioorthogonal reactivity of surface-engineered cancer cells and NK cells was assessed following modification with lipid-N_3_ or lipid-DBCO, respectively. Briefly, 1 × 10^6^ cancer or N_3_-cancer cells were incubated with 10 μM DBCO-Fluor 488 at RT for 30 min. Similarly, 1 × 10^6^ NK or D-NK cells were incubated with 10 μM N_3_-Fluor 488 under the same conditions. The cells were washed twice with serum-free medium and subsequently analyzed by flow cytometry and fluorescence microscopy. To further assess the biorthogonal click reaction activity under high-serum containing conditions, 1 × 10^6^ A549 cancer cells were first coated with 0.75 mg/ml lipid-N_3_ as described in the “Surface engineering and characterization of cancer cells and NK cells” section. The resulting N_3_-A549 cells were then incubated with 10 μM DBCO-Fluor 488 in media containing 0% to 50% FBS for 30 min at RT. Following washing, the surface-associated fluorescence intensity of DBCO-Fluor 488 on N_3_-A549 cancer cell was quantified by flow cytometry to evaluate click reaction efficiency under high-serum conditions.

### Analysis of immunological synapse formation between NK cells and target cancer cells

To evaluate immunological synapse formation between NK cells and cancer cells, F-actin staining was performed to visualize cytoskeletal organization at the NK–tumor cell interface [25]. NK cells and A549 cancer cells were first labeled with 10 μM CellTracker Blue (Invitrogen) and 1 μM Calcein-AM (Invitrogen), respectively, by incubation at 37 °C for 30 min, followed by washing twice. Subsequently, blue-labeled NK cells and green-labeled cancer cells were surface engineered with 0.75 mg/ml lipid-DBCO and 0.75 mg/ml lipid-N_3_ biomaterials, respectively, as previously described. Equal numbers of NK cells (5 × 10^4^ in 100 μl) and cancer cells (5 × 10^4^ in 100 μl) were then co-cultured at an effector-to-target (E:T) ratio of 1:1 at 37 °C for 1 h to allow NK–tumor cell direct contact and synapse formation. Following co-culture, the resulting E:T clusters were gently transferred onto poly-D-lysine (Gibco, A3890401)-coated confocal dishes and incubated for 10 min to allow cell attachment. The attached cells were subsequently fixed with 4% paraformaldehyde for 15 min at RT and washed twice with PBS. Fixed cells were permeabilized using 0.1% Triton X-100 in 1% bovine serum albumin solution for 30 min at RT. After washing, cells were incubated with Alexa Fluor 555-conjugated phalloidin (1:200 dilution; Invitrogen A34055) for 30 min at RT to visualize F-actin within the E:T clusters. Fluorescence images of NK–tumor cell clusters and F-actin organization were acquired using a fluorescence microscope. For quantitative analysis, regions of interests were defined at the direct contact interfaces between NK cells and tumor cells within E:T clusters, and the fluorescence intensity of F-actin within these contact regions was measured by ImageJ. F-actin quantification was restricted specifically to the NK–tumor contact interfaces.

### DBCO-N_3_ click reaction-mediated target recognition ability

The target recognition ability of the D-NK cells was evaluated by an E:T cluster formation assay [7]. Target cancer cells (A549 and H460) and normal lung fibroblasts (MRC-5) were stained with 1 μM CellTracker Red dye (Invitrogen) at 37 °C for 30 min, and washed twice with serum-free medium. NK cells were labeled with 0.1 μM Calcein-AM at 37 °C for 30 min, followed by 2 washes. The labeled NK cells and target cells were subsequently coated with lipid-DBCO or lipid-N_3_, respectively, as previously described. Effector NK cells and target cancer cells were co-cultured at an E:T ratio of 1:1 at 37 °C for 30 min to allow interactions. The E:T cluster formation was quantified by flow cytometry based on the double-positive population representing green-stained NK cells interacting with red-stained target cells.

### Quantification of cytokines and lytic granules

To quantify the secretion of cytokines and lytic granules, NK or D-NK cells (1 × 10^5^ cells) were co-cultured with A549 or H460 cells at a 10:1 E:T ratio at 37 °C for 4 h. Following incubation, the supernatants were collected by centrifugation. The secreted levels of cytokines (interferon-gamma [IFN-𝛾] and tumor necrosis factor-alpha [TNF-α]) and lytic granules (perforin and granzyme B) were measured using enzyme-linked immunosorbent assay (ELISA) kits (PeproTech, and Abcam) and calculated from ELISA standard curves generated using standards in the kits, according to the manufacturer’s instructions.

### Quantification of anticancer efficacy

DBCO-N_3_ click reaction-mediated anticancer efficacy was assessed by calcein release assay [26,27]. Target cancer cells (A549 and H460), normal lung fibroblasts (MRC-5), and normal endothelial cells (HUVEC) were labeled with 15 μM calcein-AM (Invitrogen) at 37 °C for 30 min and washed. Target cells were further coated with lipid-N_3_. Subsequently, 1 × 10^4^ target cells were co-incubated with NK or D-NK cells at 10:1 E:T ratios at 37 °C for 4 h. Released calcein from dead target cells was then measured using SpectraMax iD3 (excitation/emission = 485/535 nm). To calculate the specific lysis percentage, 2 control groups were established: a spontaneous release group (consisting only of target cells) and a maximum release group (100% lysed target cells achieved by treatment with 5% Triton X-100 for 30 min). The percent of specific cell lysis was calculated using the following formula:

$$\text{Specific cell lysis}\;(\%)=\frac{\left(\text{Test release}\right)-\left(\text{Spontaneous release}\right)}{\left(\text{Maximum release}\right)-\left(\text{Spontaneous release}\right)}\times 100$$ (1)

Furthermore, NK cells and target cells were coated with nonreactive lipid analogs without DBCO or N_3_ functional groups to evaluate the effect of lipid-mediated membrane insertion independent of bioorthogonal click reactions.

### Fabrication of an in vivo-mimicking 3D lung tumoroid model

A549 cells were pre-stained with 2 μM of CellTracker Red dye at 37 °C for 30 min and washed. Labeled cells were seeded into 1.5% (w/v) agarose-coated 96-well plates at a density of 2.5 × 10^3^ cells per well and centrifuged at 300×*g* for 5 min to promote cell aggregation and tumoroid formation. The plates were incubated at 37 °C in a humidified incubator with 5% CO_2_ for 3 d to allow the spontaneous formation of compact tumoroids. To recapitulate the complex structural features of lung tissue [28], the preformed A549 tumoroids were embedded within a 3D type I collagen hydrogel (2 mg/ml; Corning). Briefly, the collagen mixture was neutralized on ice, gently mixed with the tumoroid, and dispensed into 48-well plates. Gelation was achieved by incubation at 37 °C for 30 min, resulting in a stable 3D collagen hydrogel [21].

### Evaluation of 3D tumoroid labeling and anticancer performances

To evaluate the tumoroid-labeling capability of lipid biomaterials, tumoroid-containing 3D collagen gel was treated with either lipid-CFL or lipid-N_3_ biomaterial. The coating solution (0.375 mg/ml) was added directly to the tumoroid-containing collagen gel and incubated at 37 °C for 30 min to facilitate lipid diffusion and anchoring onto the tumor cell membranes. Following incubation, the gels were washed twice with serum-free medium and maintained in serum-free medium for 24 h to ensure the complete removal of unlabeled lipids. Subsequently, 5 × 10^4^ of NK or D-NK cells (equivalent of 20:1 E:T ratio) were added to tumoroid-embedded collagen gel, followed by incubation for 24 h. Tumoroid morphology and surface localization of lipid biomaterials were examined using fluorescence microscopy or confocal laser scanning microscopy (ZEISS LSM 980). To quantify NK cell-mediated cytotoxicity, the relative fluorescence intensity of CellTracker Red-labeled tumoroids remaining within the collagen gel was measured and analyzed following co-culture.

### Statistical analysis

All quantitative data are presented as the mean ± standard deviation (SD) and analyzed using unpaired Student’s *t* test or one-way analysis of variance (ANOVA) with Tukey’s multiple comparison test. Statistical significance was established at *P* values of <0.05. Statistical analyses were performed using GraphPad Prism v.10.4 (GraphPad Software Inc., USA).

## Results and Discussion

### Synthesis and characterization of the lipid-N_3_ and lipid-DBCO

We hypothesized that the membrane-inserting properties of lipid-based biomaterials could be leveraged to functionalize cancer cells and NK cells with bioorthogonal reactive moieties (i.e., azide and DBCO) to enable click chemistry-based immune recognition. As the strain-promoted azide-alkyne cycloaddition proceeds efficiently under physiological conditions without catalysts, this reaction is widely used for various cell surface engineering [29]; we envisioned that lipid-N_3_-labeled cancer cells could be selectively recognized by lipid-DBCO-engineered D-NK cells, thereby stabilizing IS and enhancing NK cell-mediated cytotoxicity.

Figure 2 illustrates the chemical synthesis of lipid-N_3_ and lipid-DBCO biomaterials, while Figs. S1 to S5 present the ^1^H-NMR spectra of all intermediates and the final lipidic conjugates. The synthesis involved sequential steps: (a) DSPE-PEG-amine was first converted to DSPE-PEG-Gly-Di-amine; (b) DSPE-PEG-Gly-Di-amine was subsequently transformed into DSPE-PEG-Gly-Di-COOH (Fig. 2A); (c) azide-PEG-amine was conjugated to the Di-COOH groups to yield DSPE-PEG-Gly-Di-PEG-azide (i.e., lipid-N_3_) biomaterial (Fig. 2B); (d) DSPE-PEG-Gly-Di-COOH was alternatively reacted with bis-PEG-amine to form DSPE-PEG-Gly-Di-PEG-amine intermediate (Fig. 2C); and in the final step, (e) DSPE-PEG-Gly-Di-PEG-amine intermediate was converted to DSPE-PEG-Gly-Di-PEG-DBCO (i.e., lipid-DBCO) via DBCO-acid conjugation (Fig. 2D). All chemical conversions were confirmed by ^1^H-NMR analysis, as shown in Figs. S1 to S5, where Fig. S3 shows that the ^1^H-NMR spectrum (500 MHz, DMSO-d_6_) of the lipid-N_3_ biomaterial exhibits characteristic peaks corresponding to DSPE terminal methyl protons (δ 0.85 ppm), lipid methylene chain protons (δ 1.49 and 2.17 to 2.61 ppm), PEG repeating units (δ 3.50 ppm), and amide bond protons (δ 7.66 to 8.21 ppm). Proton integration analysis, based on the DSPE terminal methyl protons at δ 0.85 ppm (6H) relative to PEG methylene protons at δ 3.50 ppm (556H), further confirms the successful synthesis of the lipid-N_3_ biomaterial. The successful synthesis of lipid-N_3_ and lipid-DBCO was further confirmed by FTIR spectroscopy, which showed characteristic absorption peaks corresponding to PEG chains, lipid alkyl chains, and azide or DBCO functional groups (Figs. S6 and S7).

### Generation and characterization of lipid-biomaterial-coated cancer and NK cells

**Fig. 3.**
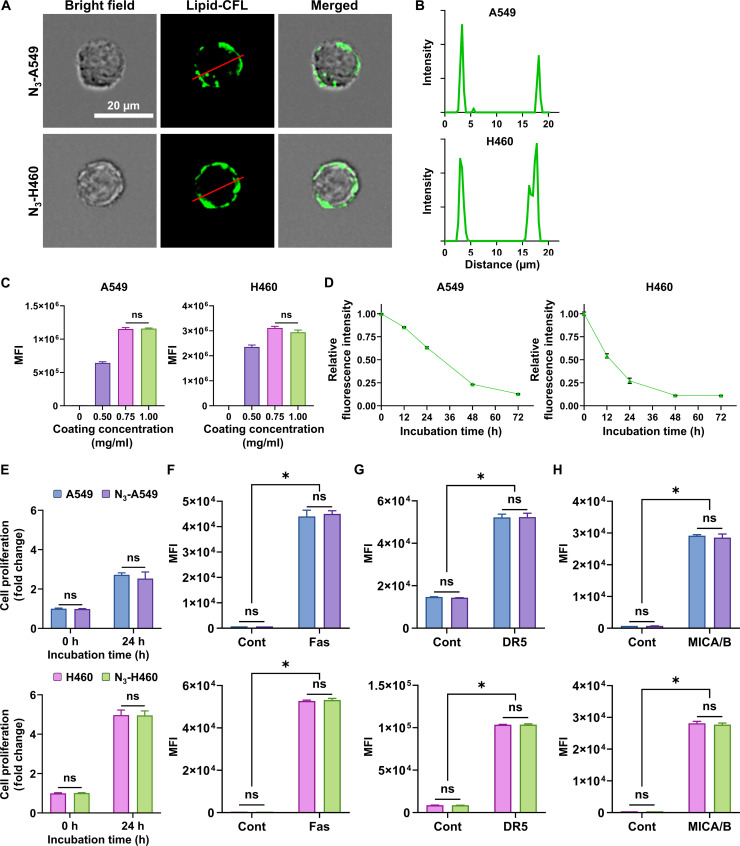
Generation and characterization of N_3_-cancer cells. (A) Optical and fluorescence microscopy images of NSCLC cells coated with lipid-5-carboxyfluorescein (lipid-CFL), demonstrating uniform membrane-immobilized coating materials. (B) Intensity profile plot of the membrane-localized distribution of the lipid biomaterials. (C) Quantitative analysis of mean fluorescence intensity (MFI) of surface coating materials at varying concentrations, assessed by flow cytometry. (D) Transient coating retention profile of the surface-immobilized lipid biomaterials. (E) Viability and proliferation capacity of the surface-engineered cancer cells. Protein expression levels of the cancer cell surface receptors (F) Fas, (G) DR5, and (H) MICA/B. Unless otherwise noted, cells were incubated with lipid-N_3_ at 0.75 mg/ml, corresponding to 0.75 mg per 5 × 10^6^ cells. All data represent the mean ± SD (*n* = 3). Statistical significance was determined by unpaired Student’s *t* test or one-way analysis of variance (ANOVA) followed by Tukey’s multiple comparison test. Differences were considered statistically significant at *P <0.05. “ns” indicates statistically nonsignificant.

Herein, we employ a lipid-biomaterial platform to enable simple and rapid ex vivo surface engineering of both cancer cells and NK cells. These lipid-based biomaterials are readily inserted into plasma membranes through hydrophobic interactions between the lipid tail and the phospholipid bilayer, enabling non-genetic modification across diverse cell types [16,17]. To confirm effective membrane insertion, we incubated NSCLC cells (A549 and H460) with lipid-CFL for 30 min at RT. Fluorescence microscopy revealed uniform membrane-associated labeling without detectable intracellular fluorescence, indicating that rather than undergoing endocytosis, the lipid-CFL integrates into the plasma membrane (Fig. 3A and B). Flow cytometric quantification further demonstrated the dose-dependent incorporation of lipid-CFL onto the cancer cell membrane, reaching a plateau at 0.75 mg/ml (Fig. 3C). Accordingly, this concentration was selected as the optimal coating condition for all subsequent experiments.

To evaluate whether membrane-anchored lipid biomaterials exhibit sufficient stability to support NK–tumor cell interactions, we characterized the membrane retention kinetics of lipid-CFL on A549 and H460 cells under physiological conditions. Based on fluorescence measurements of lipid-CFL extracted from cell lysates and normalized using a standard curve, the initial membrane-associated lipid biomaterial amounts on A549 and H460 cells were estimated to be 45.42 and 26.77 ng per 10^5^ cells, respectively (Fig. S8). Figure 3D shows that the membrane-immobilized lipid-CFL gradually dissociated from the cell surfaces of both A549 and H460 cells, with 85% and 54% of the initial fluorescence retained at 12 h, respectively. This retention time is sufficient for interaction with blood circulating D-NK cells, given that NK–tumor cell engagement occurs within the order of minutes to hours in vivo [30]. The gradual loss of lipid-anchored biomaterials from the plasma membrane is consistent with the previously reported behaviors of lipid-PEG conjugates [14,26,31]. The entry or exit of lipid-PEG anchors proceeds through the transient formation (or disruption) of a localized hydrophobic environment within the bilayer, a process that is enhanced when shorter or less hydrophobic acyl chains are used [32,33]. Hydrophobic insertion of lipid anchors is intrinsically weak and dynamic due to membrane fluidity, resulting in colloidal-like behavior in aqueous environments. Interactions with surrounding molecules, particularly serum proteins, perturb this balance and ultimately destabilize lipid–bilayer associations. For example, previous studies demonstrated that serum proteins, including lipoproteins and albumin, could extract lipid-PEG molecules from membranes through interactions with the lipid-PEG conjugates (presumably by differentiating colloidal dynamics), leading to the time-dependent depletion of surface-inserted lipids [34,35]. We further evaluated the effect of serum proteins on the membrane-anchoring efficiency of lipid biomaterials under physiologically relevant serum-rich conditions [36]. As shown in Fig. S9, increasing serum concentrations partially reduced lipid membrane anchoring, likely due to interactions with serum proteins that can mediate lipid extraction from the cell membrane. Nevertheless, a substantial fraction of lipid biomaterials remained associated with cancer cell membranes even under high-serum conditions. These results indicate that lipid biomaterials can still be effectively inserted into cancer cell membranes in serum-containing environments, supporting their potential to reinforce NK–tumor cell interactions.

Another contributing mechanism is the dilution of membrane-inserted lipids during cell proliferation, as membrane expansion and cytokinesis redistribute the initially inserted lipid moieties [22]. Similar to this mechanism, the more proliferative H460 cancer cell exhibited faster loss of lipid-CFL, compared to A549 cells (Fig. 3D and E), while exhibiting no influence on the viability and proliferative capacity of both surface-modified A549 and H460 cancer cells with 0.75 mg/ml lipid-N_3_ (Fig. 3E and Fig. S10).

We next evaluated whether lipid biomaterials affect membrane ligand/receptor availability on both NK and cancer cells. Effective engagement with immune effector cells requires the intact presentation of surface receptors on tumor cells. The anticancer efficacy of NK cells relies on ligand–receptor interactions, such as FasL–Fas, TRAIL–DR5, and NKG2D–MICA/B, to trigger extrinsic apoptosis and functional activation [37,38]. Therefore, maintaining receptor availability after lipid-mediated cell surface engineering is essential to preserve downstream immune responsiveness. Flow cytometric analysis showed that lipid-N_3_ modification did not alter the accessibility level of Fas, DR5, or MICA/B on either NSCLC cell line (Fig. 3F to H). These findings indicate that lipid-N_3_ can safely label tumor cell membranes without disrupting critical immunoregulatory receptors, supporting its suitability for applications with the preservation of NK–tumor cell interaction and effective immune activation.

**Fig. 4.**
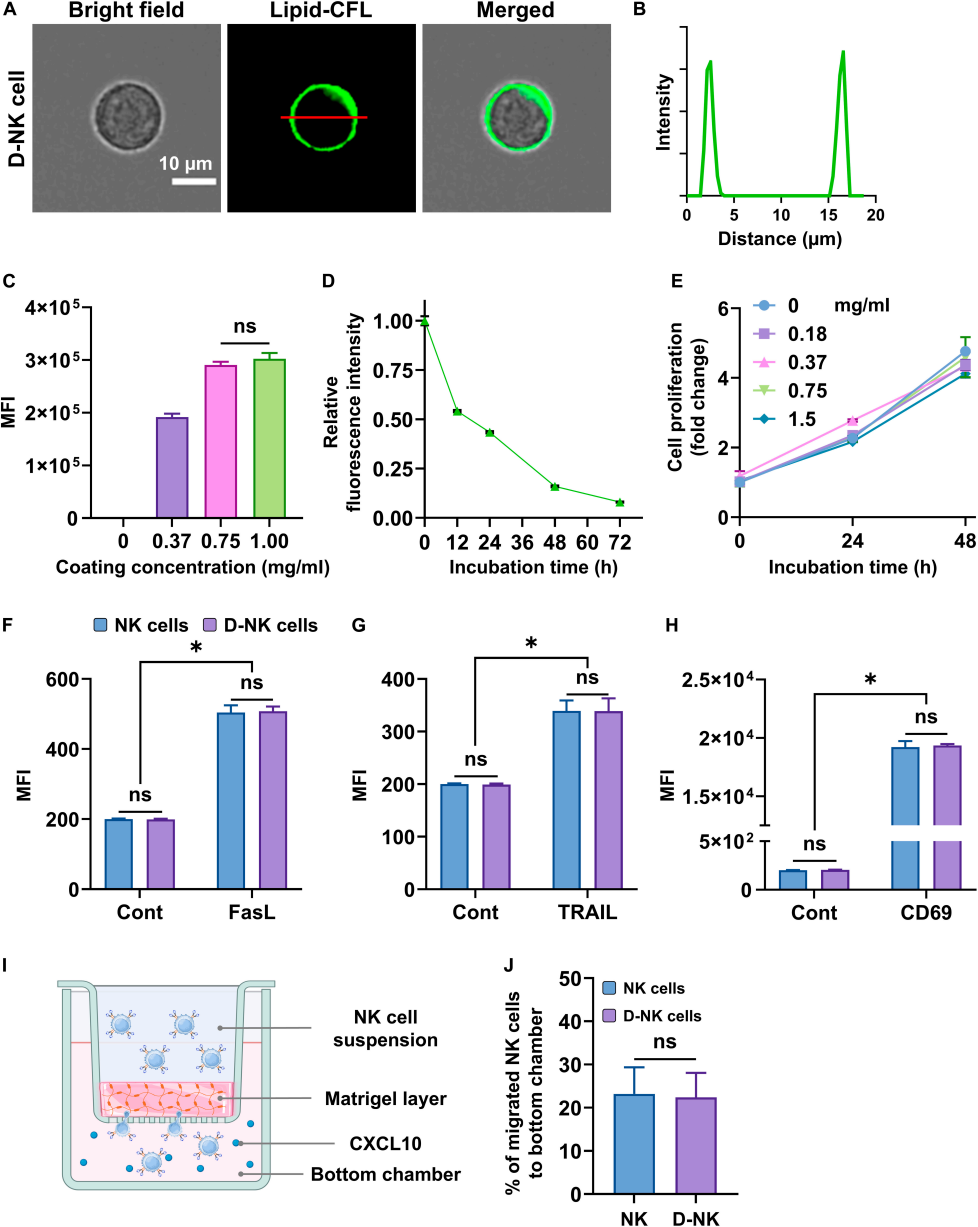
Generation and characterization of D-NK cells. (A) Optical and fluorescence microscopy images of NK cells coated with lipid-CFL. (B) Intensity profile plot of D-NK cell surfaces. (C) Flow cytometric MFI analysis across varying coating concentrations. (D) Retention kinetics of lipid biomaterials at NK cell membranes. (E) Viability and proliferative capacity of surface-engineered NK cells. Protein expression levels of NK cell surface ligands/receptors (F) Fas ligand (FasL), (G) tumor necrosis factor-related apoptosis-inducing ligand (TRAIL), and (H) CD69 after surface engineering. (I) Schematic of the designed transwell migration assay used to evaluate NK cell migration. The transwell insert was coated with Matrigel to mimic the physical barrier of the tumor. CXCL10 was used as a chemoattractant for NK cells. (J) The percentage of migrated NK cells to the bottom chamber after 24 h of incubation. Unless otherwise noted, NK cells were incubated with lipid-DBCO at 0.75 mg/ml, corresponding to 0.75 mg per 5 × 10^6^ cells. All data represent the mean ± SD (*n* = 3). Statistical significance was determined by unpaired Student’s *t* test or one-way ANOVA followed by Tukey’s multiple comparison test. Differences were considered statistically significant at *P < 0.05. “ns” indicates statistically nonsignificant.

To enable DBCO-N_3_ click reactions at the NK–cancer cell interfaces, cancer cells were engineered with lipid-N_3_ and NK cells with lipid-DBCO, creating complementary membrane chemistries that were able to reinforce the IS formation upon contact. D-NK cells were generated and systematically characterized using an analytical workflow parallel to that used for N_3_-labeled cancer cells. Fluorescence imaging of lipid-CFL-modified NK cells showed membrane-confined signal, indicating efficient insertion into the NK cell membrane, without detectable intracellular accumulation (Fig. 4A and B). The flow cytometry analysis shown in Fig. 4C further reveals the dose-dependent membrane labeling, while identifying 0.75 mg/ml as the optimal coating concentration, above which fluorescence intensity plateaus, indicating the saturation of the membrane-insertion capacity. Under these optimized conditions, the amounts of membrane-inserted lipid biomaterials were estimated to be approximately 59.18 ng per 10^5^ NK cells (Fig. S8). The stability of the membrane-inserted lipid biomaterials was further evaluated under physiological culture conditions (Fig. 4D). Notably, more than 54% of the lipid biomaterial was retained on the NK cell surface over 12 h, a sufficient time with dynamic NK–tumor cell surveillance, synapse formation, and effector activation processes in vivo, as also shown in our previous investigations [7,26].

D-NK cells preserved their viability and proliferative capacity across a wide concentration range of 0.18 to 1.5 mg/ml, confirming that neither the lipid biomaterial itself nor the membrane-insertion process imposed measurable cytotoxic or proliferative deficits (Fig. 4E). Importantly, the major cytotoxic effector ligands on NK cells (FasL and TRAIL) and activation receptor (CD69) remained accessible following surface modification with lipid-DBCO (Fig. 4F to H). The maintained expression levels indicate that immobilization of lipid-DBCO onto NK cell membranes does not sterically interfere with ligand–receptor interactions required for NK cell-mediated extrinsic apoptosis, nor does it induce nonspecific activation of NK cells [39]. We also evaluated the expression levels of NK cell membrane components at 48 h after lipid-DBCO coating. Surface-engineered D-NK cells exhibited comparable expression levels of membrane-associated molecules including FasL, TRAIL, and CD69, relative to unmodified NK cells (Fig. S11). These results suggest that lipid-DBCO-mediated NK cell surface engineering preserves the accessibility of NK cell membrane components without inducing unintended activation. Furthermore, for translational applicability, NK cells should migrate toward tumor sites to induce therapeutic effects. To assess whether lipid-DBCO biomaterials affect NK cell motility, a transwell migration assay was performed using inserts coated with Matrigel to recapitulate the physical barriers of the TME (Fig. 4I). As shown in Fig. 4J, 23.2% of NK cells and 22.4% of D-NK cells migrated toward the CXCL10 gradient [24], indicating no significant difference in migratory capacity.

Collectively, these results establish that D-NK cells can be robustly generated while preserving viability, receptor accessibility, effector functionality, and motility. The biocompatibility and stability of lipid-DBCO anchoring support the potential of a promising strategy to (a) engineer NK cells to form covalent conjugation with N_3_-labeled cancer cells and (b) enhance IS formation through bioorthogonal click chemistry.

### Bioorthogonal reactivity at the cell membrane interfaces

To validate the bioorthogonal functionality of lipid-anchored reactive groups at the NK–cancer interface, we assessed whether lipid-DBCO-coated NK (D-NK) cells and lipid-N_3_-engineered cancer (N_3_-cancer) cells retained membrane-confined reactivity following surface engineering. Lipid-PEG-based biomaterials are known to spontaneously incorporate into the plasma membranes through hydrophobic interactions while presenting their functional moieties outward, enabling controlled surface functionalization without genetic modification [22,40]. Based on previous reports, we hypothesized that lipid-N_3_ and lipid-DBCO would (a) stably anchor to cancer and NK cell membranes, respectively, and (b) maintain selective reactivity toward their partner functional groups, enabling DBCO-N_3_ click conjugation upon NK–tumor contact.

**Fig. 5.**
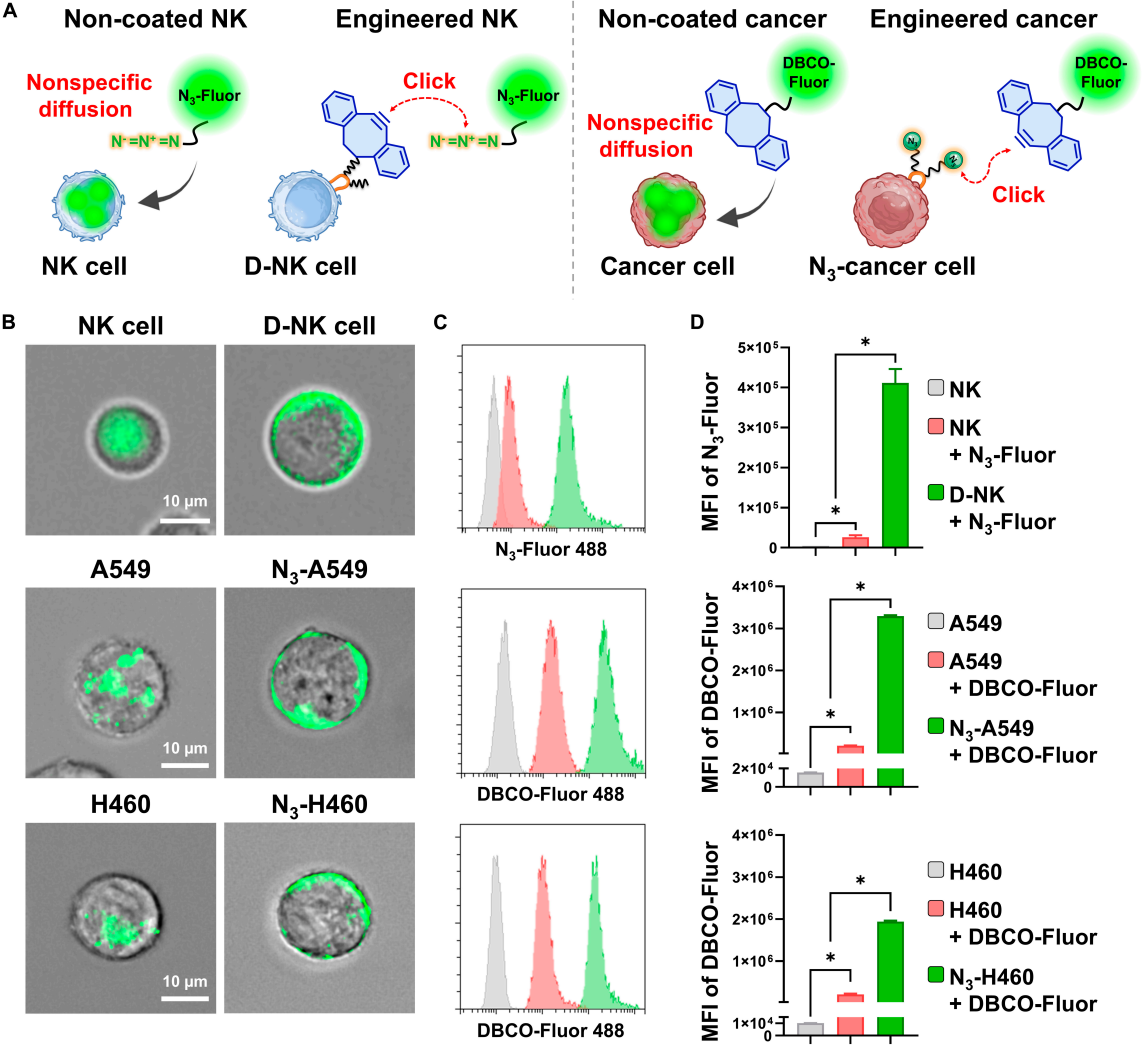
Bioorthogonal reactivity on cell surfaces. (A) Schematic of the DBCO-N_3_ click reaction at cell surfaces. (B) Fluorescence microscopy images of D-NK or N_3_-cancer cells labeled with N_3_-Fluor or DBCO-Fluor. (C) Representative histogram plots and (D) MFI of NK or cancer cells treated with N_3_-Flour or DBCO-Flour. All data represent the mean ± SD (*n* = 3). Statistical significance was determined by one-way ANOVA followed by Tukey’s multiple comparison test. Differences were considered statistically significant at *P < 0.05.

To test this hypothesis, D-NK cells were incubated with N_3_-Fluor 488 dye, while N_3_-cancer cells were incubated with DBCO-Fluor 488 dye for 30 min at RT (Fig. 5A). Non-coated NK and cancer cells served as controls. In Fig. 5B, fluorescence microscopy revealed that non-engineered NK and cancer cells exhibited diffuse intracellular fluorescence upon incubation with N_3_-Fluor or DBCO-Fluor, which is attributed to nonspecific cellular uptake of excess fluorescence dye. In contrast, D-NK cells displayed predominantly membrane-localized fluorescence signals following incubation with N_3_-Fluor 488, while N_3_-cancer cells similarly showed strong peripheral fluorescence upon treatment with DBCO-Fluor 488, indicating selective labeling at the cell surface. Consistently, the MFI of N_3_-Fluor or DBCO-Fluor was significantly increased in surface-engineered NK and cancer cells, indicating the successful click reaction at the cell membranes (Fig. 5C and D). We further evaluated the bioorthogonal reactivity of tumor cell membrane-anchored lipid-N_3_ under physiologically relevant high-serum conditions. Given that tumor cell surface-immobilized lipid-N_3_ should retain its functionality prior to interaction with circulating D-NK cells, we assessed the reaction between lipid-N_3_ and DBCO-Fluor 488 in serum-rich conditions that mimic the protein-dense environment of blood [36]. As shown in Fig. S12, the DBCO-N_3_ click-reaction efficiency was partially attenuated in the presence of serum. This reduction is likely attributable to nonspecific interactions between serum proteins and hydrophobic moieties, which can competitively hinder bioorthogonal conjugation [29]. Despite this attenuation, a substantial fluorescence signal remained detectable on N_3_-A549 cells following incubation with DBCO-Fluor 488, indicating that membrane-anchored lipid-N_3_ retains sufficient accessibility and reactivity under serum-rich conditions. These results confirm that our material-mediated ex vivo surface engineering successfully inserts the functionalized amphiphiles into the cell membrane, while maintaining the accessibility and reactivity of the DBCO and azide groups. This is consistent with prior studies demonstrating that cell membrane-decorated biomaterials maintain bioorthogonal reactivity on live-cell membranes and support diverse applications, such as drug delivery [41], cell–cell tethering [15], and in vivo bioimaging [42]. The membrane-restricted fluorescence observed here demonstrates that both lipid-DBCO and lipid-N_3_ resist nonspecific internalization and remain chemically competent at the extracellular interface.

Overall, these results confirm that both D-NK cells and N_3_-cancer cells retain fully functional bioorthogonal groups at their membrane interfaces, enabling DBCO-N_3_ click reactivity upon direct cell–cell physical engagement. This membrane-localized reactivity provides the mechanistic foundation for stable intercellular interaction between NK cells and tumor targets, supporting lipid-biomaterial-mediated surface engineering as an effective strategy to reinforce NK–tumor synapse formation and promote selective immune engagement.

### Reinforcement of NK–tumor cell engagement mediated by DBCO-N_3_ click reaction

**Fig. 6.**
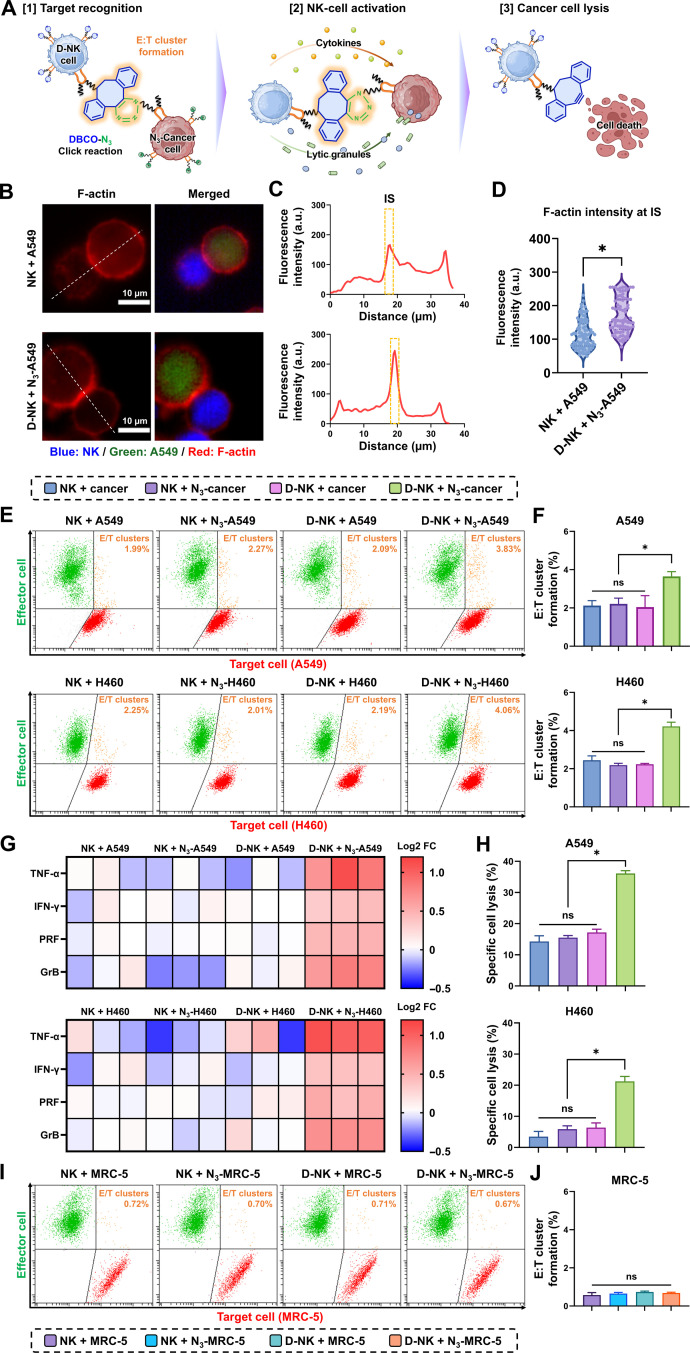
In vitro anticancer efficacy facilitated by DBCO-N_3_ click reaction. (A) Schematic of the target recognition and activation process of NK cells through click reaction. (B) Representative images of IS formation between NK cells and target cancer cells. (C) Line profile analysis of fluorescence intensity across NK–tumor interfaces, indicating localized F-actin enrichment at the IS. (D) Quantification of F-actin fluorescence intensity at NK–tumor cell contact interfaces (*n* = 91 and *n* = 82). (E and F) Quantification of effector-to-target ratio (E:T) cluster formations between NK and cancer cells. (G) Secretion levels of pro-inflammatory cytokines (tumor necrosis factor-alpha [TNF-α] and interferon-gamma [IFN-γ]) and lytic granules (perforin [PRF] and granzyme B [GrB]) by NK cells, following co-culture with A549 or H460 cells. Cytokine and lytic granule concentrations were quantified using enzyme-linked immunosorbent assay and calculated from standard curves generated with standards provided in the kits. FC, fold change. (H) Specific lysis of target cancer cells by NK and D-NK cells after 4 h of co-culture with A549, H460, or N_3_-cancer cells at a 10:1 E:T ratio. Tumor-selective E:T clustering assessed in normal lung fibroblasts (MRC-5), shown by (I) representative dot plots and (J) quantification of E:T cluster formation. Data represent the mean ± SD (*n* = 3). Statistical significance was determined by unpaired Student’s *t* test or one-way ANOVA followed by Tukey’s multiple comparison test. Differences were considered statistically significant at *P < 0.05. “ns” indicates statistically nonsignificant.

Although various strategies have been developed to enhance antitumor efficacy within immunosuppressive TMEs, including biomaterial-mediated metabolic reprogramming, combinatorial therapies that induce immunogenic cell death, and approaches to alleviate tumor hypoxia [43–46], the present study focuses on an alternative strategy that directly enhances physical interactions between NK cells and cancer cells. NK cell cytotoxicity is governed by a stepwise process that involves initial target recognition, followed by activation and IS maturation (Fig. 6A) [47]. Physical contact between NK cells and their targets initiates synapse assembly, which subsequently enables the directed secretion of cytokines and lytic granules responsible for tumor cell elimination. Prior studies have shown that the enhanced targeting ability of NK–tumor interfaces could substantially improve cytotoxic performance, particularly in solid tumors with poor immunological contact [7,26]. Here, we reinforced these contact-dependent processes using a membrane-immobilized bioorthogonal conjugation system. Membrane-immobilized reactive groups (DBCO on NK cells and N_3_ on cancer cells) enable rapid strain-promoted azide-alkyne cycloaddition upon cell–cell physical contact. Unlike native receptor–ligand interactions, which are reversible, DBCO-N_3_ reactions proceed with high-rate constants (1 to 60 M^−1^·s^−1^) under physiological conditions without the need for catalytic species [29]. This DBCO-N_3_ reaction is expected to increase the effective avidity of the NK–tumor cell interactions and prolong contact duration, thereby stabilizing IS formation at the cellular interfaces, without interfering with endogenous ligand–receptor signaling.

NK cell-mediated cytotoxicity critically depends on the formation of an IS, a specialized intercellular junction characterized by dynamic cytoskeletal reorganization [48]. In particular, F-actin polymerization and accumulation at the NK–target cell interface are well-established hallmarks of IS formation, playing essential roles in synapse initiation, stabilization, and functional maturation [25]. To evaluate IS formation between NK cells and cancer cells, F-actin staining was performed to visualize cytoskeletal organization at the NK–tumor cell interface. As shown in Fig. 6B and Fig. S13, fluorescence imaging revealed the formation of NK–tumor cell conjugates accompanied by localized F-actin accumulation at the contact interfaces. Line profile analysis further demonstrated characteristic actin enrichment at the NK–tumor interfaces (Fig. 6C). Notably, the D-NK + N_3_-A549 group exhibited significantly higher F-actin intensity at these contact interfaces compared with the unmodified NK + A549 group (Fig. 6D). To further systematically assess the contribution of DBCO-N_3_ click chemistry to NK cell–tumor recognition, co-culture assays were conducted using NSCLC cell lines (A549 and H460) and normal lung fibroblasts (MRC-5). Target recognition was quantified by measuring E:T cluster formation at 30 min after co-incubation. Figure 6E and F show that robust and stable E:T cluster formation was obtained exclusively when D-NK cells encountered N_3_-cancer cells, whereas both NK + N_3_-cancer cells and D-NK + unmodified cancer cells exhibited no significant increases over the control NK–cancer interaction, indicating that the augmented recognition response was not driven by nonspecific adhesion, but required complementary DBCO and N_3_ presentation at opposing cell membranes.

To determine whether this enhanced physical engagement translated into downstream NK activation, NK cells were co-cultured with target cells at a 10:1 E:T ratio for 4 h, and the secretion of effector cytokines (TNF-α and IFN-γ) and lytic granules (perforin and granzyme B) was quantified by ELISA. NK cells activate upon target recognition and release these molecules to mediate apoptosis and amplify local immune responses [47]. Consistent with the recognition assay (Fig. 6E and F), only the interactions of D-NK + N_3_-cancer cells elicited a significant increase in cytokine and granule secretion, whereas the other groups displayed secretion profiles comparable to the unmodified NK–cancer group (Fig. 6G and Fig. S14). This pattern indicates that NK activation was specifically induced by DBCO-N_3_ click-mediated synapse stabilization. In consequence, the D-NK + N_3_-cancer group exhibited significantly elevated specific lysis of both A549 and H460 cells, compared to all other groups (Fig. 6H). Neither D-NK cells alone nor N_3_-engineered cancer cells alone improved killing efficiency, again demonstrating that therapeutic enhancement required the presence of both reactive groups and the ensuing click reaction. Furthermore, when NK or cancer cells were coated with nonreactive lipid analogs (i.e., lipid biomaterial without DBCO or N_3_ functional groups), no enhancement of NK cell-mediated cytotoxicity against either A549 or H460 cancer cells was observed (Fig. S15). These results confirm that the enhanced anticancer activity of D-NK cells toward N_3_-cancer cells is specifically mediated by bioorthogonal DBCO-N_3_ click chemistry at the NK–tumor cell interfaces. Importantly, both NK and D-NK cells exhibited negligible clustering with N_3_-modified control lung fibroblasts, indicating minimal off-target effects on normal cells (Fig. 6I and J). These results demonstrate that surface-anchored DBCO and N_3_ moieties cooperatively promote selective and robust physical engagement between NK cells and NSCLC cells. Furthermore, D-NK cells showed no detectable cytotoxicity toward N_3_-coated normal lung fibroblasts and human endothelial cells (Fig. S16), confirming that bioorthogonal conjugation did not compromise innate NK selectivity, which was mediated by self-tolerance mechanism [17].

Taken together, these results demonstrate that the DBCO-N_3_ click reaction serves as an important mediator of enhanced NK–tumor cell recognition, activation, and tumor-specific cytotoxicity. Mechanistically, these bioorthogonal interactions enhance NK cell function through (a) increasing the effective avidity of NK–tumor cell interactions and (b) prolonging the duration of cell–cell contact, thereby facilitating stable IS formation. This is supported by enhanced F-actin accumulation and polarization at the NK–tumor interface, indicative of improved synapse maturation, as well as increased secretion of cytokines and lytic granules. Collectively, these findings indicate that DBCO-N_3_ click chemistry functions to stabilize NK–tumor cell engagement, thereby strengthening tumor cell lysis while maintaining target specificity and minimizing off-target interactions.

### DBCO-N_3_ click reaction-mediated anticancer functionality in biomimetic 3D collagen gel

Figures 3 and 5 show that lipid biomaterials enabled efficient and uniform surface immobilization on cancer cells at the single-cell level. A brief 30-min incubation with lipid-CFL or lipid-N_3_ resulted in membrane-confined fluorescence signals without detectable intracellular accumulation, confirming the spontaneous insertion of lipid anchors into the plasma membrane. This efficient surface engineering provided a stable platform to present bioorthogonal functional groups on living cancer cells, while preserving the availability of cell membrane receptors. Consequently, the engineered cancer cell membrane interface (functionalized with N_3_ moiety) served as a chemically addressable reaction site that was able to selectively engage complementary moieties on immune cell surfaces (functionalized with DBCO moiety). Leveraging these engineered membrane interfaces, we successfully confirmed that bioorthogonal coupling between N_3_-cancer cells and D-NK cells could potentiate intercellular interactions and immune effector function (Fig. 6).

**Fig. 7.**
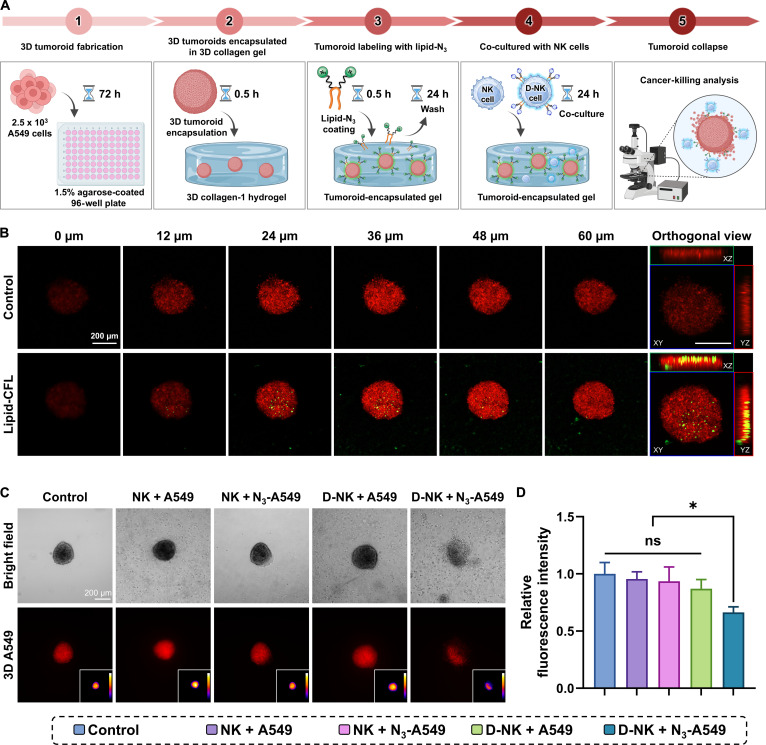
Lipid-biomaterial-mediated labeling of 3D lung tumoroids and DBCO-N_3_ click reaction-driven anticancer functionality of D-NK cells in a biomimetic 3D collagen matrix. (A) Schematic of 3D tumoroid formation, surface labeling with lipid biomaterials, and subsequent co-culture with NK or D-NK cells within a type I collagen hydrogel. (B) Representative confocal microscopic images showing the surface immobilization of lipid-CFL on 3D tumoroids embedded in a collagen matrix, demonstrating effective lipid diffusion and membrane anchoring within a biomimetic TME. (C and D) Representative fluorescence microscopy images and quantitative analysis of tumoroid disruption, indicated by a reduction in CellTracker Red fluorescence, following 24 h co-culture with NK or D-NK cells after lipid-N_3_ labeling. Data represent the mean ± SD (*n* = 3). Statistical significance was determined by one-way ANOVA followed by Tukey’s multiple comparison test. “*P < 0.05” indicates significant differences between the D-NK + N_3_-A549 group and the other experimental groups. “ns” indicates nonsignificant differences.

To evaluate the performance of lipid-mediated surface engineering under physiologically relevant conditions, we employed a 3D collagen hydrogel-based lung tumoroid model. The collagen hydrogel (2 mg/ml) used in this study falls within the literature-reported range for modeling lung ECM environments and has been shown to exhibit mechanical properties (Young’s modulus ~2 kPa) and fibrillar architectures comparable to native lung tissue, as well as pore sizes (~8.45 ± 2.37 μm^2^) that permit cell migration [21,28]. Accordingly, this system recapitulates major ECM-mediated physical constraints, including nanofibrous architecture, diffusion limitation, and restricted cell infiltration. 3D tumoroids were generated to recapitulate the multicellular architecture of solid tumors and subsequently embedded within a collagen gel formulated to approximate the mechanical properties of native lung tissue (Fig. 7A steps 1 and 2). Compact and spherical 3D tumoroids were first fabricated and used to assess whether lipid biomaterials could uniformly decorate tumor surfaces in a multicellular architecture. Following only 30-min incubation with lipid-CFL and subsequent washing, fluorescence microscopy revealed homogeneous labeling across the surface of the 3D lung tumoroids (Fig. S17). The fluorescence signals observed on 3D tumoroids indicate that lipid-based biomaterials can rapidly and uniformly coat not only individual cells but also the surfaces of dense, multicellular tumor assemblies. These findings highlight the scalability of lipid-mediated surface engineering from single-cell to 3D tumor structures.

We then examined lipid coating performance within an even more stringent setting by embedding CellTracker Red-stained tumoroids inside a 3D collagen network (Fig. 7A step 3). Figure 7B shows that despite the presence of a complex fibrillar matrix, confocal microscopic images demonstrated clear lipid-CFL signals inserted into the surfaces of tumoroids encapsulated deep within the collagen gel. Notably, while lipid-CFL uniformly coated tumoroid surfaces under suspension conditions (Fig. S17), incomplete surface coverage was observed in collagen-embedded tumoroids, indicating restricted lipid biomaterial accessibility imposed by the ECM (Fig. 7B). Despite these limitations, a substantial fraction of lipid-CFL remained associated with tumoroid membranes, demonstrating the feasibility of lipid-mediated membrane insertion within a dense ECM environment. Importantly, even with only 30 min of exposure to collagen-embedded tumoroids, membrane-associated fluorescence signals were clearly detectable, highlighting both the tissue-penetrating capability of the lipid anchors and their rapid membrane-anchoring efficiency. Given that the average pore area of type I collagen gels at a final concentration of 2 mg/ml was reported to be 8.45 ± 2.37 μm^2^ [21], lipid biomaterials are expected to readily diffuse through the collagen matrix. This result indicates that lipid-based cancer cell surface engineering remains effective, even within dense ECM that mimics the major physical barriers of the in vivo TME. As lipid-mediated membrane insertion relies on hydrophobic interactions and is not intrinsically tumor specific, this platform may be further improved by integration with established tumor-targeting strategies to achieve enhanced cancer-selective delivery [49,50].

Having confirmed successful tumor surface labeling in the 3D collagen system, we next evaluated the augmentation of NK cell-mediated antitumor activity driven by DBCO-N_3_ click chemistry (Fig. 7A step 4). We employed a tumor-specific quantification strategy by pre-labeling tumoroids with CellTracker Red and measuring the reduction in tumor-associated fluorescence as an indicator of tumoroid viability and structural integrity, enabling selective monitoring of tumoroid-derived signals [7]. CellTracker Red pre-stained tumoroids embedded in 3D collagen gels were transiently coated with lipid-N_3_, thoroughly washed to remove unbound material, and co-cultured with either unmodified NK cells or D-NK cells at a 20:1 E:T ratio. After 24 h, quantitative analysis of residual CellTracker Red fluorescence signals revealed a pronounced reduction only in the D-NK + N_3_-tumoroid group (Fig. 7A step 5 and C and Fig. S18). Specifically, this group exhibited a 34% decrease in tumoroid-associated fluorescence relative to control tumoroids, whereas despite identical co-culture durations, no other conditions showed significant change (Fig. 7D). These findings elucidate that the DBCO-N_3_-mediated physical engagement extends beyond simplified single-cell assays to a tissue-mimetic 3D environment. Within the collagen matrix, D-NK cells were able to migrate, establish stable contact with N_3_-labeled tumoroids, and form IS that translated into enhanced tumor cell killing. The anticancer efficacy was not improved by lipid-N_3_ coating alone or by the presence of unmodified NK cells. In contrast, a significant reduction in tumoroid-associated fluorescence was observed only when D-NK cells interacted with N_3_-cancer cells, indicating enhanced tumor cell killing. These findings demonstrate that DBCO-N_3_ click chemistry-mediated reinforcement of NK–tumor cell interactions remains effective even under ECM-constrained conditions, supporting the potential applicability of this strategy in physiologically relevant TMEs.

Thus, these findings establish a conceptual framework for engineering immune cell–tumor cell interactions using lipid-based biomaterials. Lipid biomaterials enable the rapid, uniform, and membrane-confined surface modification of both single cancer cells and structurally complex 3D tumoroids. Importantly, this surface engineering capability is preserved within dense, lung-tissue-mimicking collagen matrices, demonstrating effective diffusion and stable membrane anchoring under biomimetic conditions. Notably, bioorthogonal DBCO-N_3_ click chemistry between D-NK cells and N_3_-tumor cells reinforces effector–target physical engagement, stabilizes IS formation, and amplifies antitumor activity in a physiologically relevant 3D microenvironment. Taken together, our membrane manipulation is a versatile and translatable strategy to enhance immune cell-based therapies against solid tumors by overcoming the limited targeting ability of NK cells within the TMEs.

## Conclusion

In this study, we developed lipid biomaterials comprising DBCO and N_3_ functional modules conjugated to a lipid anchor for the ex vivo cell surface engineering of both NK and cancer cells. These lipid biomaterials enable a rapid, one-step, and non-genetic surface modification strategy that simplifies the engineering process, while preserving the intrinsic biological properties of both cell types. By leveraging bioorthogonal DBCO-N_3_ click chemistry at cell membrane interfaces, this approach overcomes major technical limitations of adoptive NK cell therapy for NSCLC, including insufficient tumor-targeting specificity and limited immune activation. Notably, the bioorthogonal click reaction mediates NK–cancer cell interactions independently of endogenous ligand–receptor pairing, thereby facilitating stable IS formation between NK cells and tumor cells. Upon engagement, surface-engineered D-NK cells exhibited robust activation, as evidenced by the significantly elevated secretion of TNF-α, IFN-γ, perforin, and granzyme B, resulting in enhanced direct tumor cell lysis. Furthermore, the surface-labeling efficacy of lipid-N_3_ biomaterials was validated in 3D tumoroid models embedded within collagen hydrogels that recapitulate the complex structural features of native lung tissue. The lipid biomaterials efficiently diffused through the hydrogel matrix and coated tumoroid surfaces, demonstrating effective labeling beyond single-cell systems. Importantly, lipid-N_3_-decorated tumoroids in complex 3D collagen hydrogels were efficiently disrupted by D-NK cells, indicating that (a) lipid biomaterials can stably immobilize functional groups on tumor cell surfaces within complex TMEs and (b) membrane-immobilized DBCO-N_3_ click reaction substantially augments the anticancer activity of NK cells. Overall, this bidirectional cell surface engineering strategy establishes a lipid-mediated, surface marker-independent, chemically programmable interface to direct immune cell–tumor cell interactions. By pairing tumor cells labeled with defined chemical functionalities (i.e., azide) and NK cells bearing complementary reactive groups (i.e., DBCO), solid tumors within heterogeneous and dense TMEs can be effectively targeted and eliminated. Despite the demonstrated efficacy of this platform, lipid-mediated membrane anchoring lacks intrinsic tumor cell specificity, and serum components may partially reduce membrane-anchoring efficiency and bioorthogonal reactivity under physiological conditions. To address these limitations, future studies will focus on integrating this strategy into tumor-targeted, antifouling lipid nanoparticle systems designed to enable selective delivery while minimizing protein corona formation, thereby enhancing in vivo stability, click reaction efficiency, and overall translational potential. Beyond cancer immunotherapy, this modular and lipid-based biomaterial design provides a broadly adaptable platform to investigate and engineer cell–cell interactions, construct multilayered cellular assemblies, and enable the controlled fusion of distinct cell populations for advanced therapeutic and biofabrication applications.

## Data Availability

Data will be made available on reasonable request.
