# Supplementary material for: Click Chemistry Mediated Immune Synapse Augmentation in Natural Killer Cell–Cancer Membrane Engagement and Facilitated Anticancer Efficacies of Natural Killer Cell Therapy
Source: Biomater Res. 2026 Jun 9;30:0376. doi: 10.34133/bmr.0376 (PMC13247315; doi:10.34133/bmr.0376)
Supplement: Supplementary 1 — Figs. S1 to S18 [file bmr.0376.f1.docx]

**Supplementary Materials**

**Click Chemistry-mediated Immune Synapse Augmentation in Natural Killer Cell-Cancer Membrane Engagement and Facilitated Anticancer Efficacies of Natural Killer Cell Therapy**

Kyung Mu Noh^1^, Ashok Kumar Jangid^1^, Eunha Kim^1^, and Kyobum Kim^1,2 *^

^1^Department of Chemical & Biochemical Engineering, Dongguk University, Seoul, Republic of Korea

^2^Cellbastian Inc., Seoul, Republic of Korea

^*^ Corresponding author: Kyobum Kim

*E-mail addresses:* kyobum.kim@dongguk.edu (K. Kim)


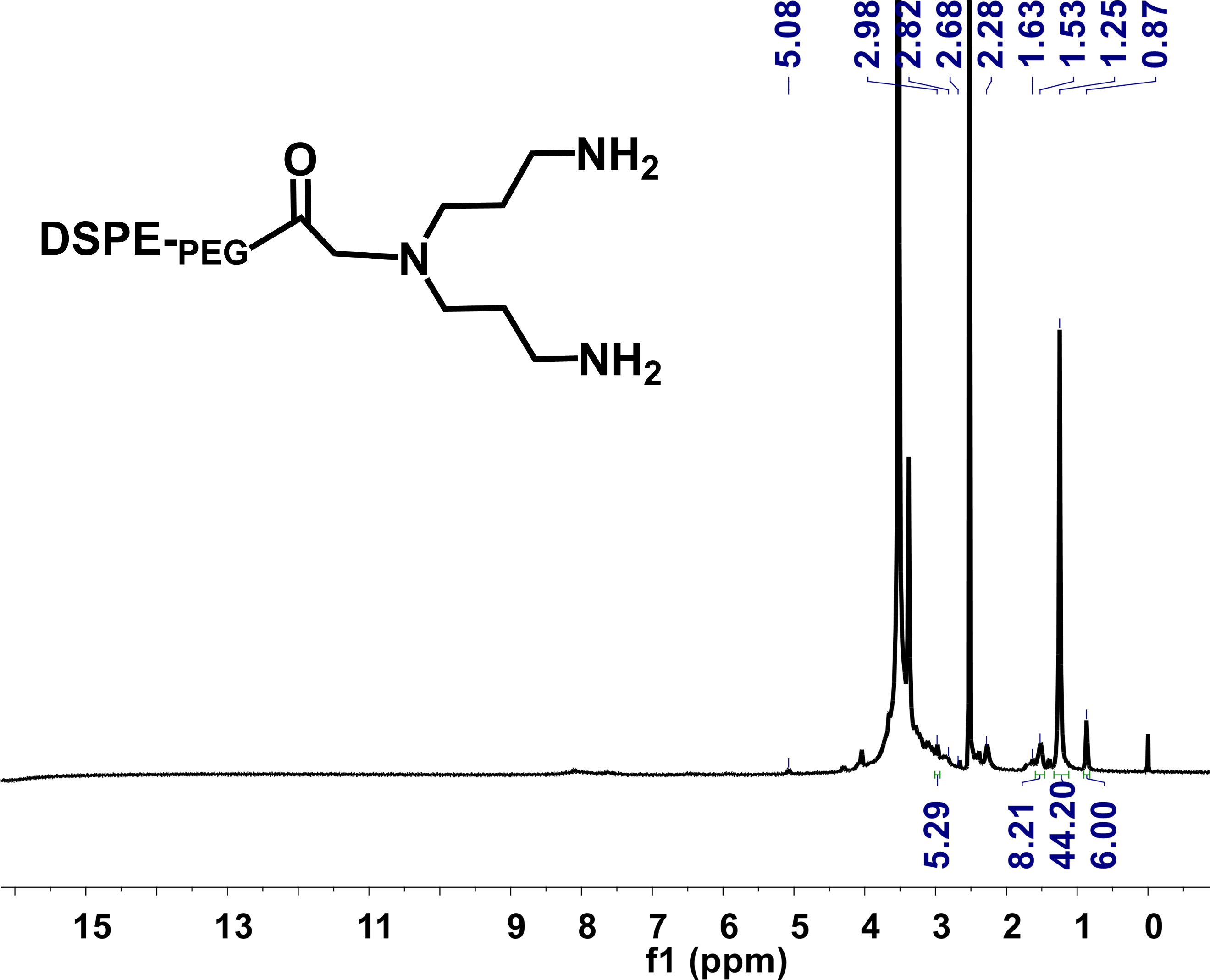


**Fig. S1. Proton NMR spectra of the synthesized DSPE-PEG-Gly-Di-amine.** ^1^H-NMR (500 MHz, DMSO) DSPE terminal methyl group (δ 0.87 ppm) and two-lipid methyl chain protons and methyl of aminopropyl moiety (δ 1.25, 1.53, 1.63, 2.28 to 2.98 ppm).


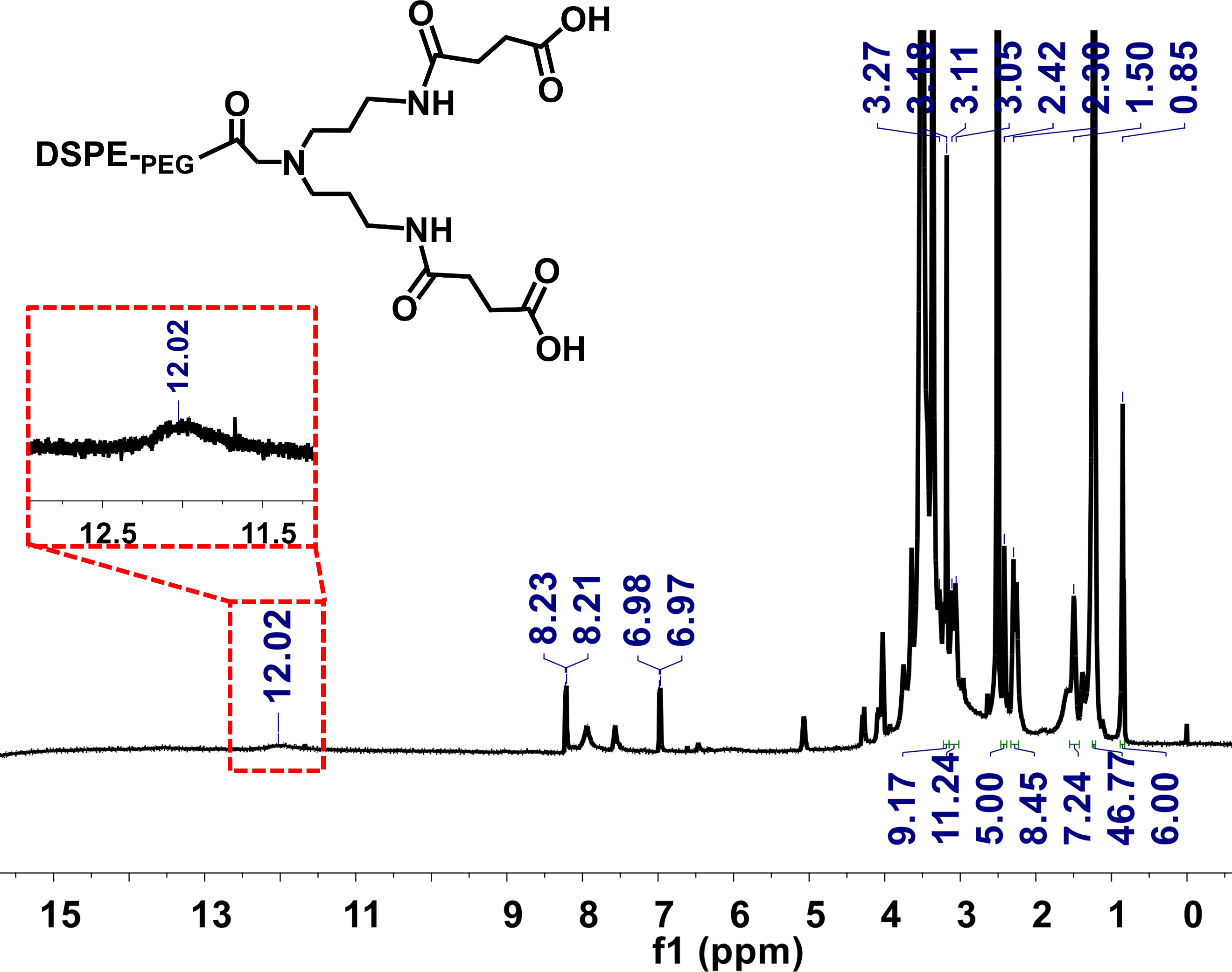


**Fig. S2. Proton NMR spectra of the synthesized DSPE-PEG-Gly-Di-COOH.** ^1^H-NMR (500 MHz, DMSO) DSPE terminal methyl group (δ 0.85 ppm), two-lipid methyl chain protons, methyl of aminopropyl moiety and succinoyl methyl group (δ 1.23, 1.50, 2.30, 2.42, 3.05, 3.11, 3.18, 3.27 ppm), amide bond protons (δ 6.97 to 8.23 ppm), and terminal COOH group protons (δ 12.02 ppm).


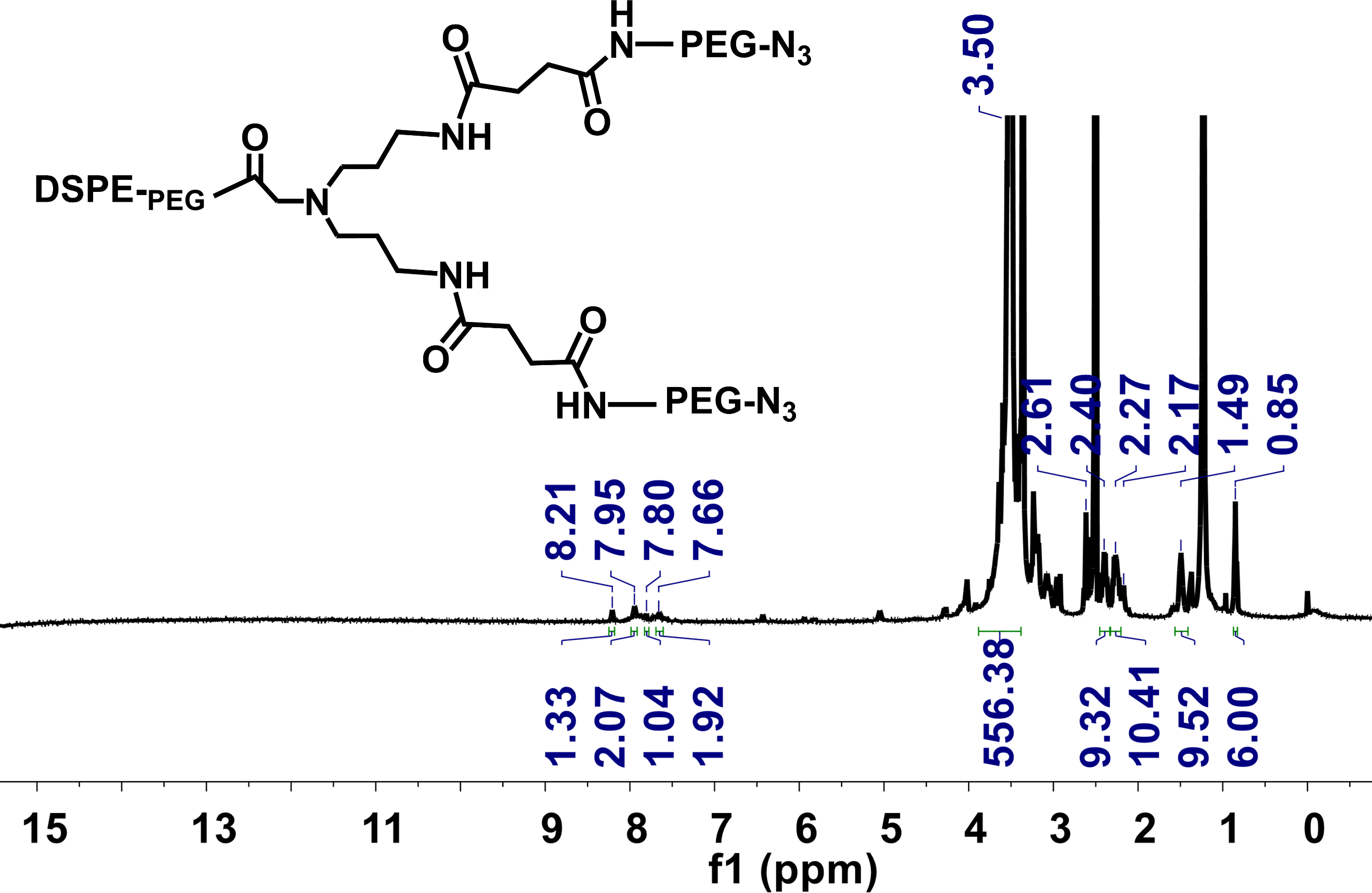


**Fig. S3. Proton NMR spectra of the synthesized DSPE-PEG-Gly-Di-PEG-azide (i.e., Lipid-N_3_) biomaterial.** ^1^H-NMR (500 MHz, DMSO) DSPE terminal methyl group (δ 0.85 ppm), two-lipid methyl chain protons (δ 1.49, 2.17 to 2.61 ppm), PEG repeating units (δ 3.50 ppm), and amide bond protons (δ 7.66 to 8.21 ppm). The proton integration analysis based on the DSPE terminal methyl protons at δ 0.85 ppm (6 H), compared to PEG protons at δ 3.50 ppm (556 H) confirms the successful synthesis of the Lipid-N_3_ biomaterial.


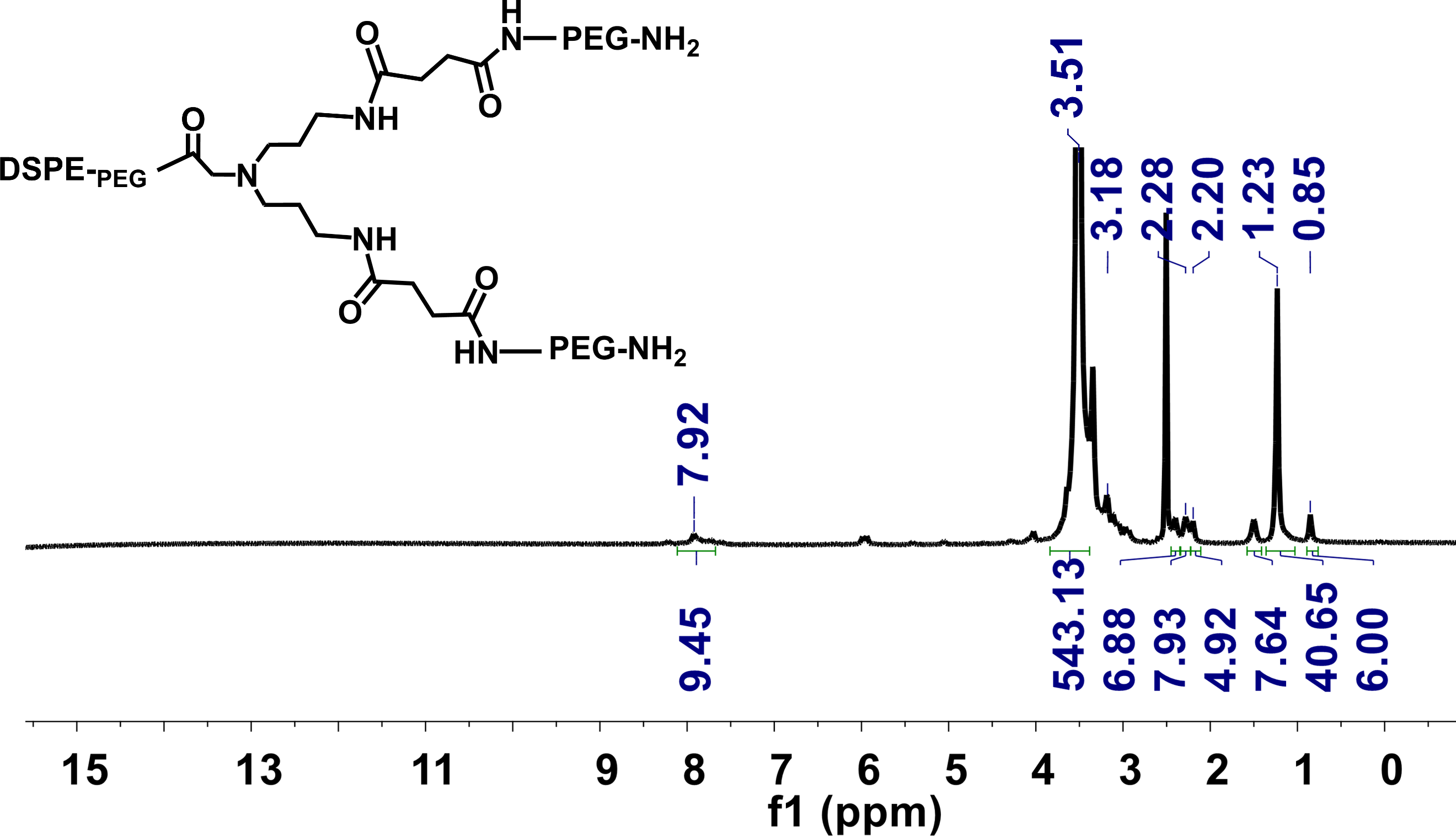


**Fig. S4. Proton NMR spectra of the synthesized DSPE-PEG-Gly-Di-PEG-amine intermediate.** ^1^H-NMR (500 MHz, DMSO) DSPE terminal methyl group (δ 0.85 ppm), two-lipid methyl chain protons (δ 1.23, 2.20 to 3.18 ppm), PEG repeating units (δ 3.51 ppm), and amide bond protons (δ 7.92 ppm). The proton integration analysis based on the DSPE terminal methyl protons at δ 0.85 ppm (6H), compared to PEG protons at δ 3.50 ppm (543 H), confirms the successful synthesis of the DSPE-PEG-Gly-Di-PEG-amine intermediate.


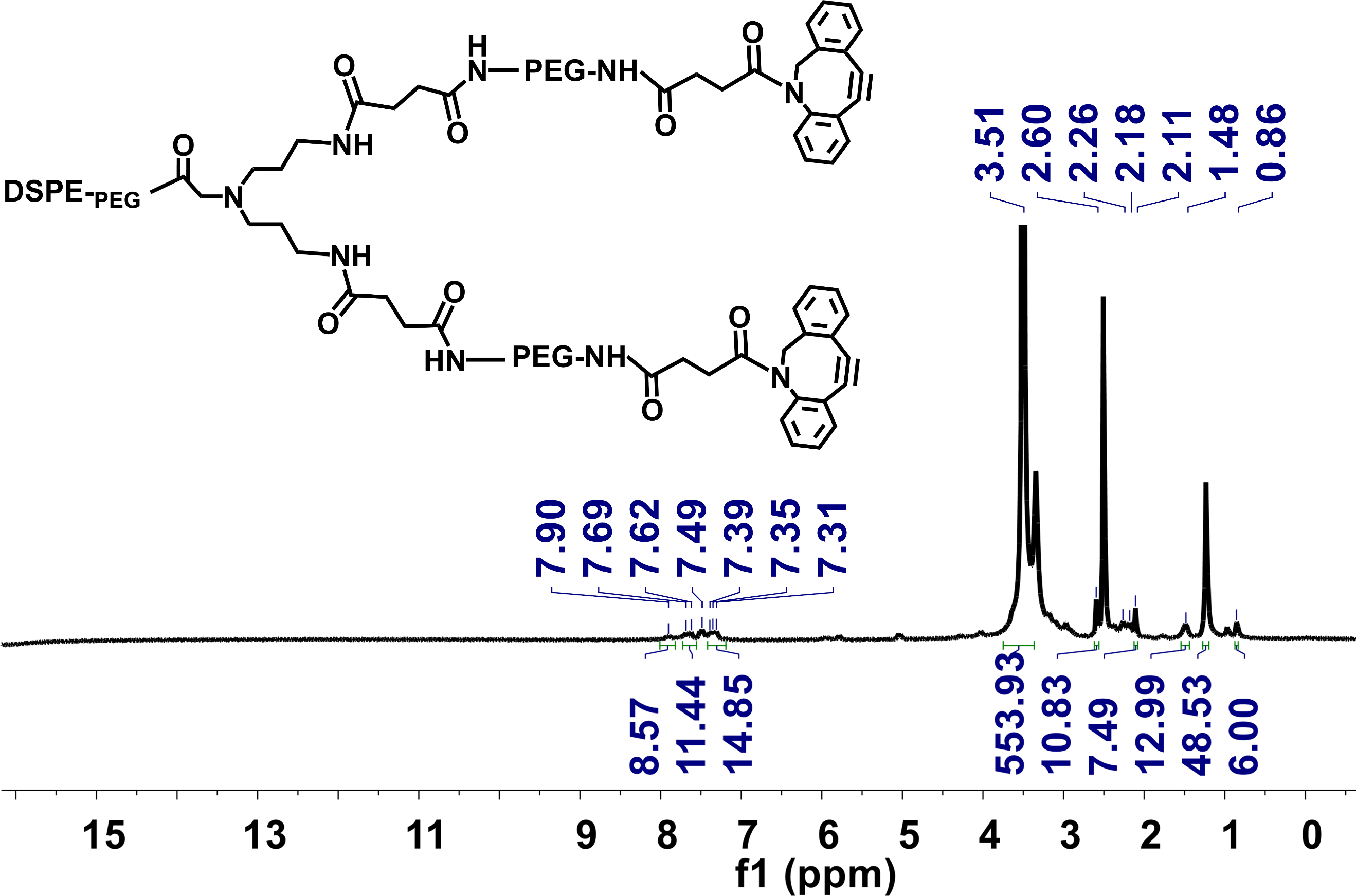


**Fig. S5. Proton NMR spectra of the synthesized DSPE-PEG-Gly-Di-PEG-DBCO (i.e., Lipid-DBCO) biomaterials.** ^1^H-NMR (500 MHz, DMSO) DSPE terminal methyl group (δ 0.86 ppm), two-lipid methyl chain protons (δ 1.48, 2.11 to 2.60 ppm), PEG repeating units (δ 3.51 ppm), and DBCO phenyl group protons (δ 7.31 to 7.90 ppm). Proton integration analysis based on the DSPE terminal methyl protons at δ 0.86 ppm (6H), compared to PEG protons at δ 3.51 ppm (553 H), along with the presence of DBCO phenyl signals, confirms the successful synthesis of the DSPE-PEG-Gly-Di-PEG-DBCO (Lipid-DBCO) biomaterial.


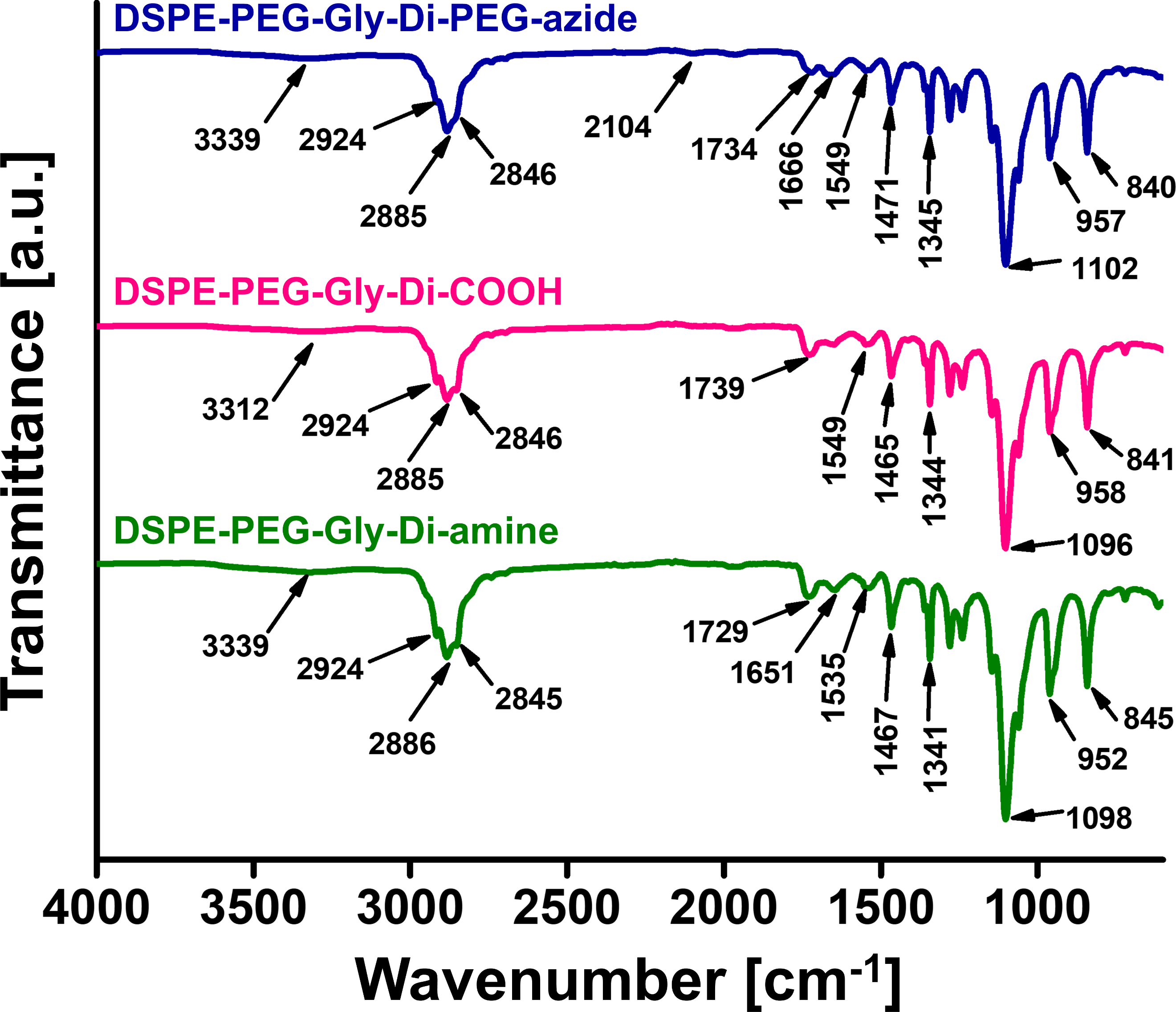


**Fig. S6. FTIR spectra of DSPE-PEG-Gly-Di-amine, DSPE-PEG-Gly-Di-COOH, and DSPE-PEG-Gly-Di-azide (i.e., Lipid-N_3_ biomaterial).** The FTIR characteristic peaks of DSPE-PEG-Gly-Di-PEG-azide shows as: PEG and DSPE-PEG N–H stretching (3339 cm^–1^), C–H stretching (2924, 2885 and 2846 cm^–1^), a small azide stretching (2104 cm^–1^), DSPE-PEG >C═O stretching (1731 cm^–1^), amide bond >C═O stretching (1666 cm^–1^), PEG and DSPE-PEG N-H and C–H bending (1549–1345 cm^–1^), and C–O stretching (1102-840 cm^–1^).


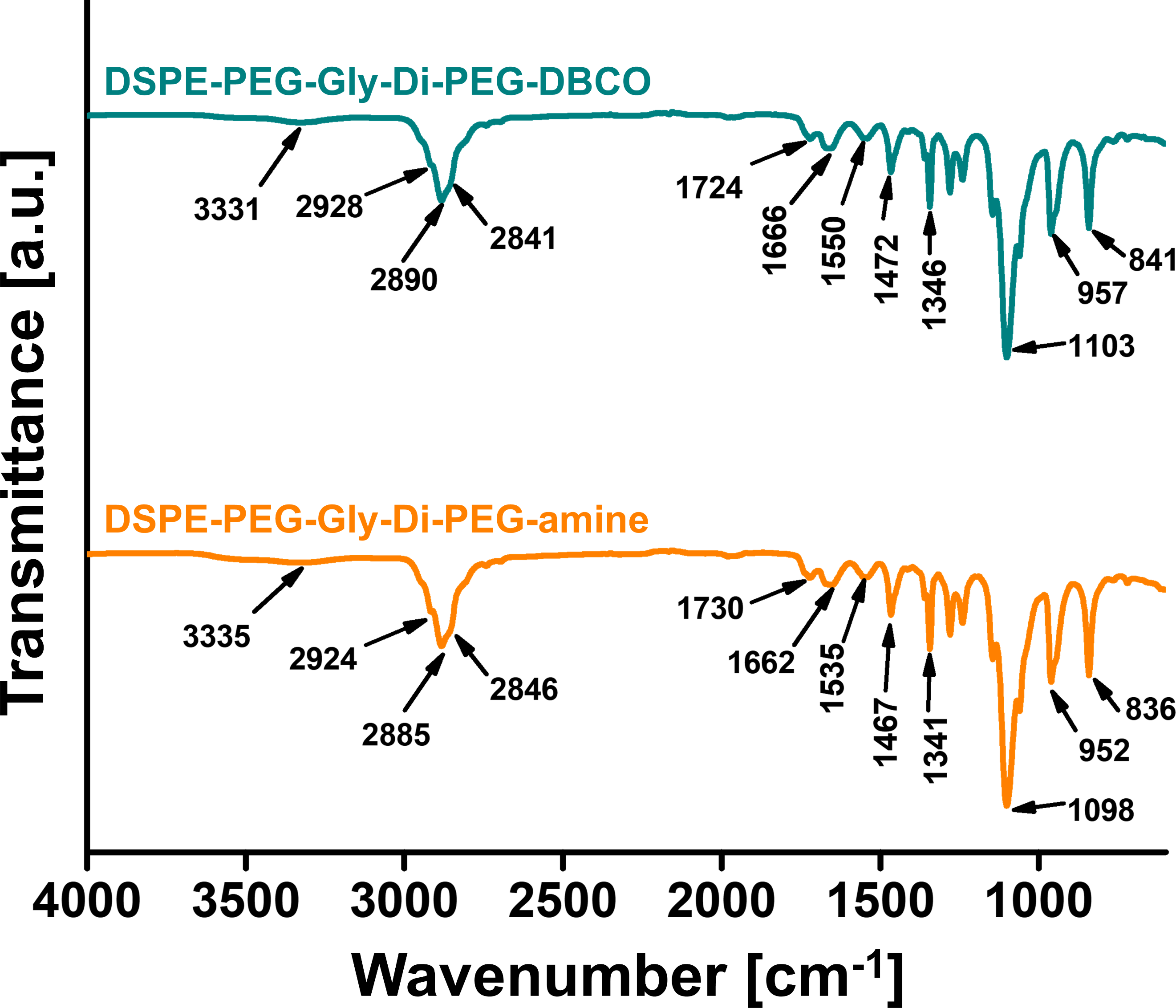


**Fig. S7. FTIR spectra of DSPE-PEG-Gly-Di-PEG-amine and DSPE-PEG-Gly-Di-PEG-DBCO (i.e., Lipid-DBCO biomaterial).** The FTIR characteristic peaks of DSPE-PEG-Gly-Di-PEG-DBCO shows as: PEG and DSPE-PEG N–H stretching (3331 cm^–1^), C–H stretching (2928, 2890 and 2841 cm^–1^), DSPE-PEG >C═O stretching (1724 cm^–1^), amide bond >C═O stretching (1666 cm^–1^), PEG and DSPE-PEG N-H and C–H bending (1550–1346 cm^–1^), and C–O stretching (1103-841 cm^–1^).


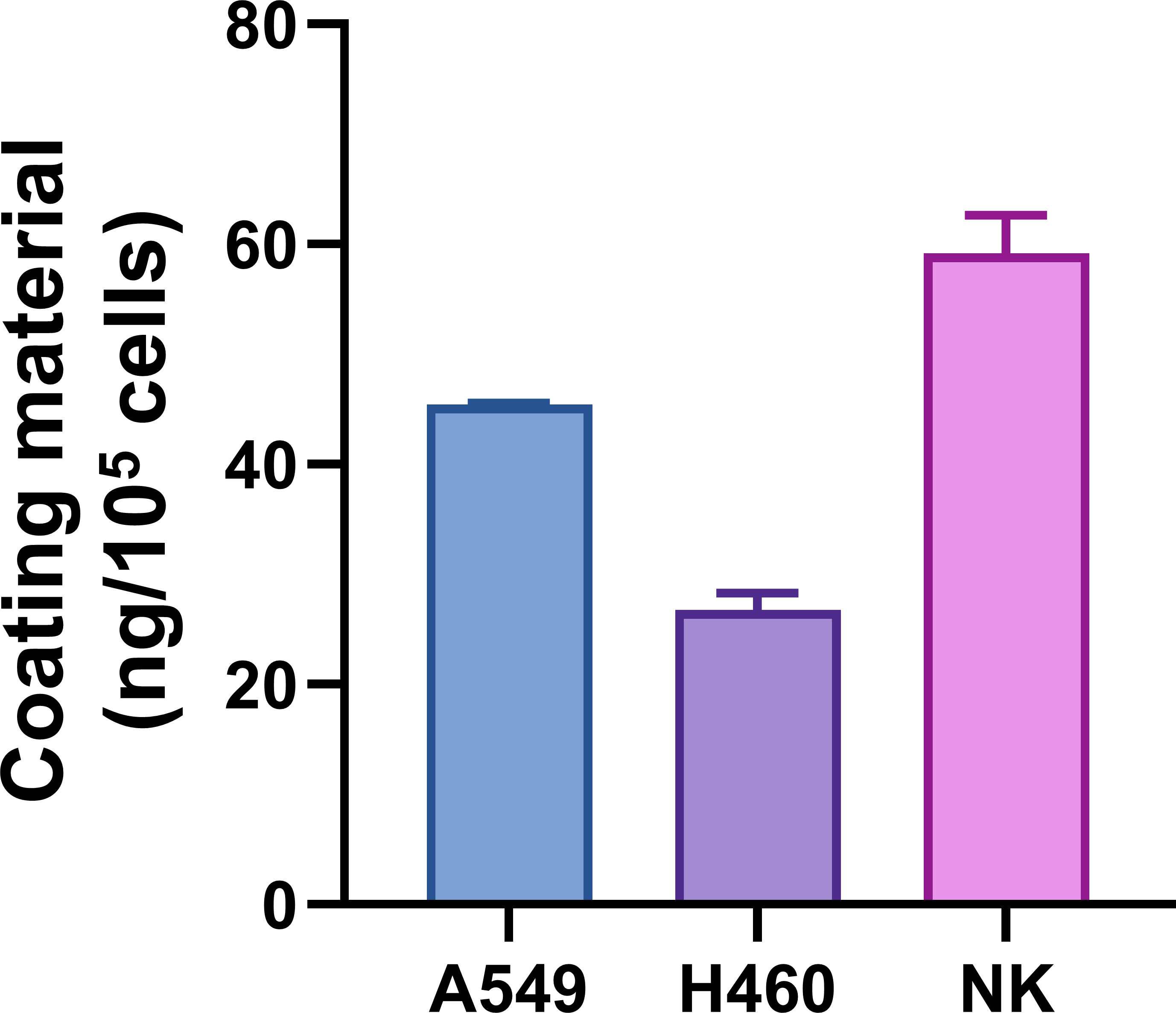


**Fig. S8. Cell surface-associated coating materials after engineering with Lipid-CFL.** The amount of cell coating materials was quantified fluorescence intensity of Lipid-CFL on cell surfaces. The amount of membrane-inserted Lipid-CFL was calculated using a standard curve generated from serial dilutions of Lipid-CFL.


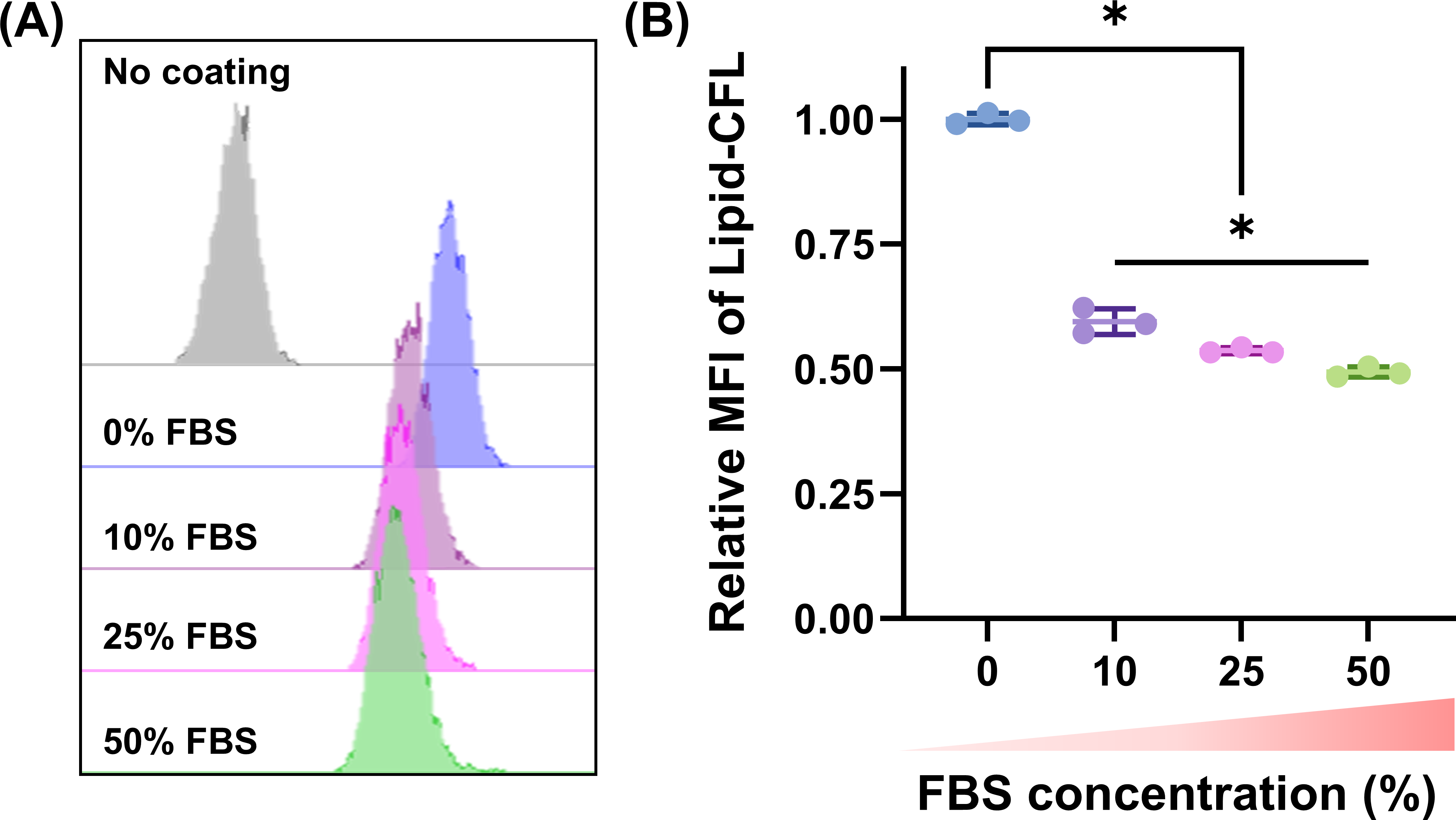


**Fig. S9. Membrane anchoring efficiency of lipid biomaterials under high serum-containing conditions.** (A) Representative histogram plots showing Lipid-CFL fluorescence on A549 cancer cells. (B) Relative MFI of Lipid-CFL on A549 cancer cells after incubation with 0.75 mg/mL Lipid-CFL in media containing 0–50% FBS. Statistical significance was determined by one-way ANOVA followed by Tukey’s multiple comparison test. Differences were considered statistically significant at **p* < 0.05.


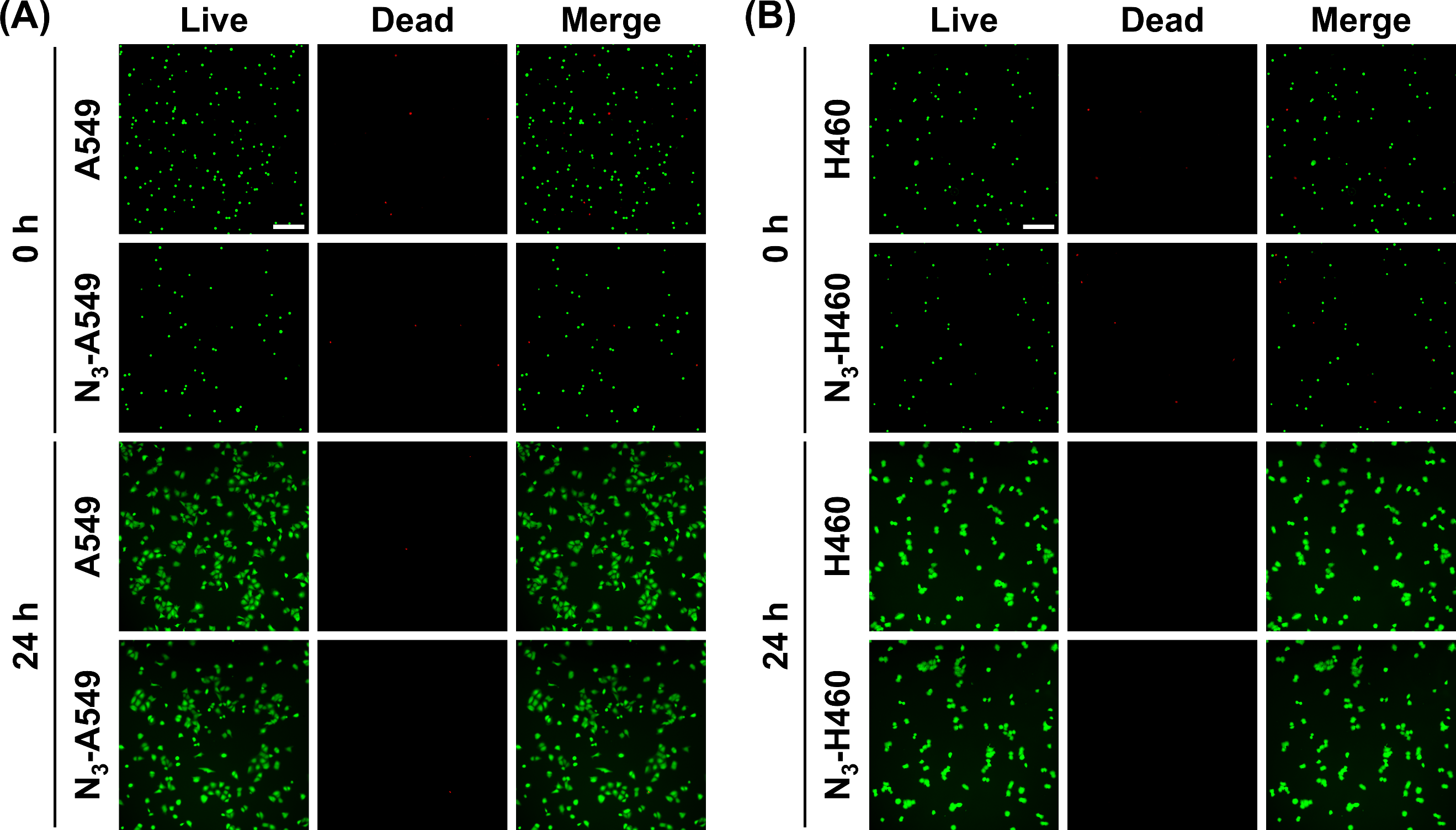


**Fig. S10. Live and dead analysis of (A) A549 and (B) H460 cells, after surface engineering with Lipid-N_3_.** The Lipid-N_3_-coated cancer cells were stained with Calcein-AM and EthD-1 solutions to evaluate cell viability at the indicated time points. (Scale bar: 200 µm).


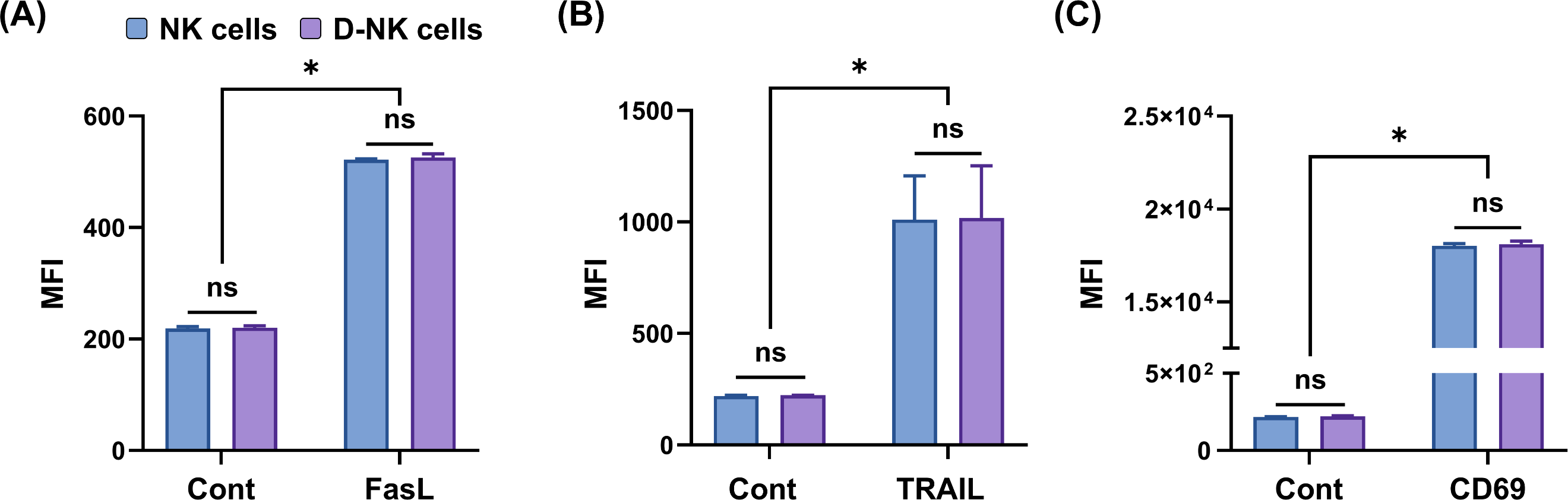


**Fig. S11. Analysis of NK cell membrane components after surface engineering with Lipid-DBCO at 48 h.** NK cells and D-NK cells were cultured NK cell growth conditions for 48 h. Then, the NK cell membrane components (A) FasL, (B) TRAIL, and (C) CD69 were analyzed through flow cytometry. Statistical significance was determined by unpaired student’s t-test or one-way ANOVA followed by Tukey’s multiple comparison test. Differences were considered statistically significant at **p* < 0.05. “ns” indicates statistically non-significant.


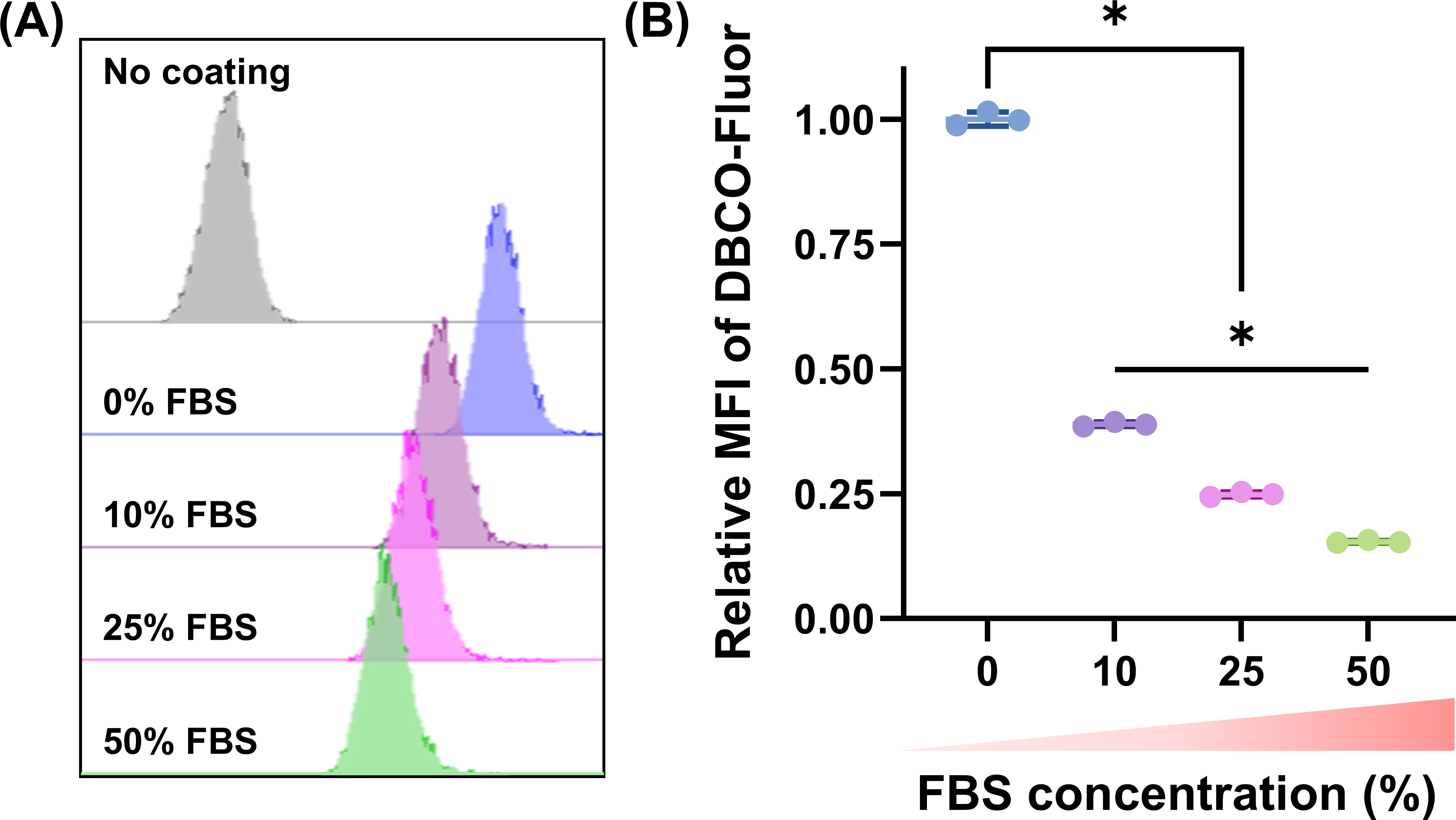


**Fig. S12. Click reaction activity on N_3_-A549 cancer cells under high serum-containing conditions.** (A) Representative histogram plots for click reaction activity. (B) N_3_-A549 cancer cells were surface engineered with 0.75 mg/mL of Lipid-N_3_ in serum-free medium. Then, N_3_-A549 cells were co-incubated with 10 µM DBCO-Fluor dye in the medium containing 0 – 50% FBS to mimic physiological serum concentrations. Statistical significance was determined by one-way ANOVA followed by Tukey’s multiple comparison test. Differences were considered statistically significant at **p* < 0.05.


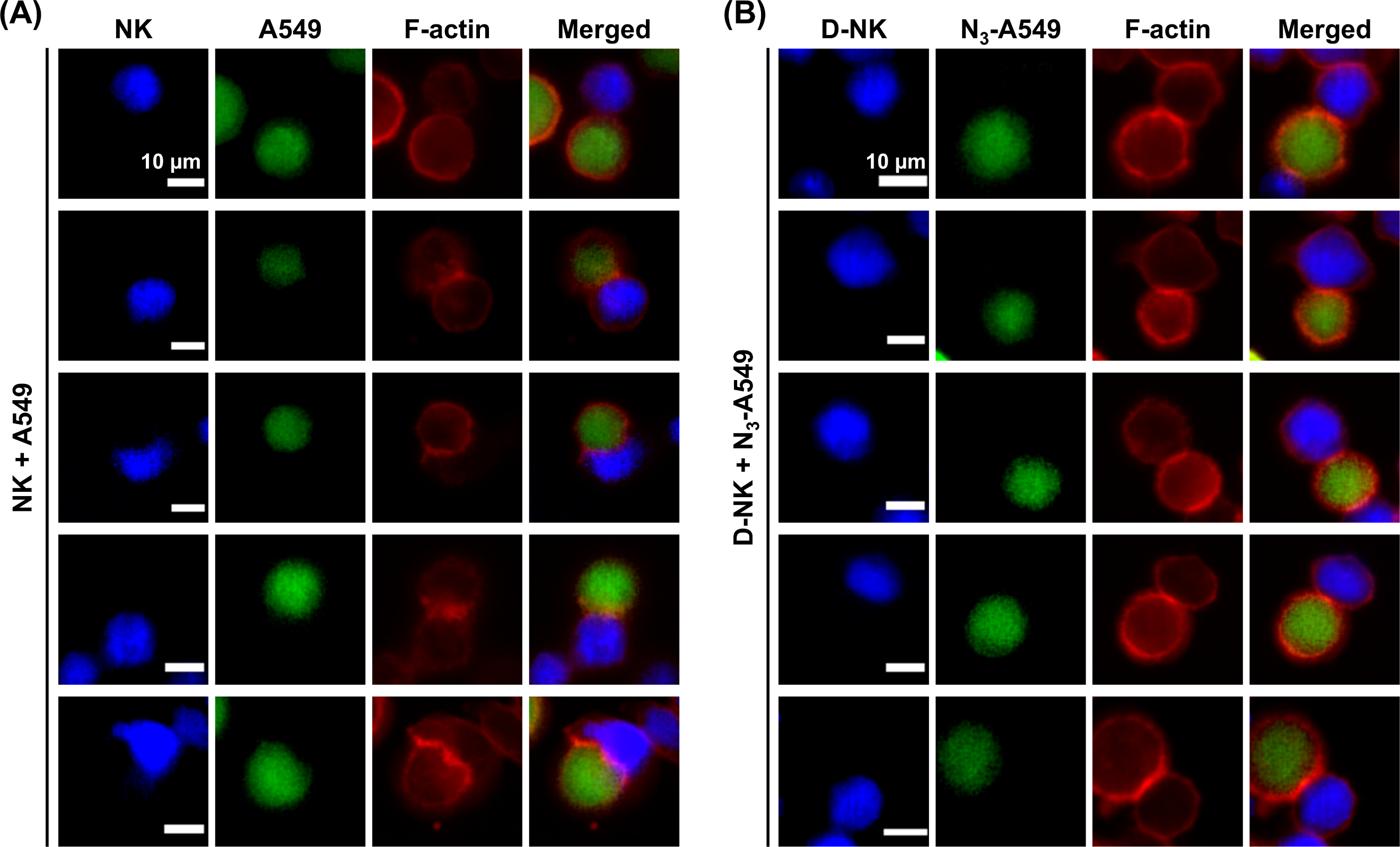


**Fig. S13. Representative fluorescence images of immunological synapse formation between NK cells and A549 cancer cells.** NK cells were pre-labeled with 10 µM CellTracker Blue, and A549 cancer cells were pre-labeled with 1 µM Calcein-AM. Subsequently, NK or Lipid-DBCO-coated NK (D-NK) cells were co-cultured with target (A) A549 cells or (B) Lipid-N3-coated A549 (N3-A549) cells for 1 h to induce immunological synapse formation. Following co-culture, NK-tumor cell clusters were seeded onto poly-D-lysine-coated confocal dishes and fixed. Immunological synapse formation was visualized by F-actin staining and analyzed using fluorescence microscopy.


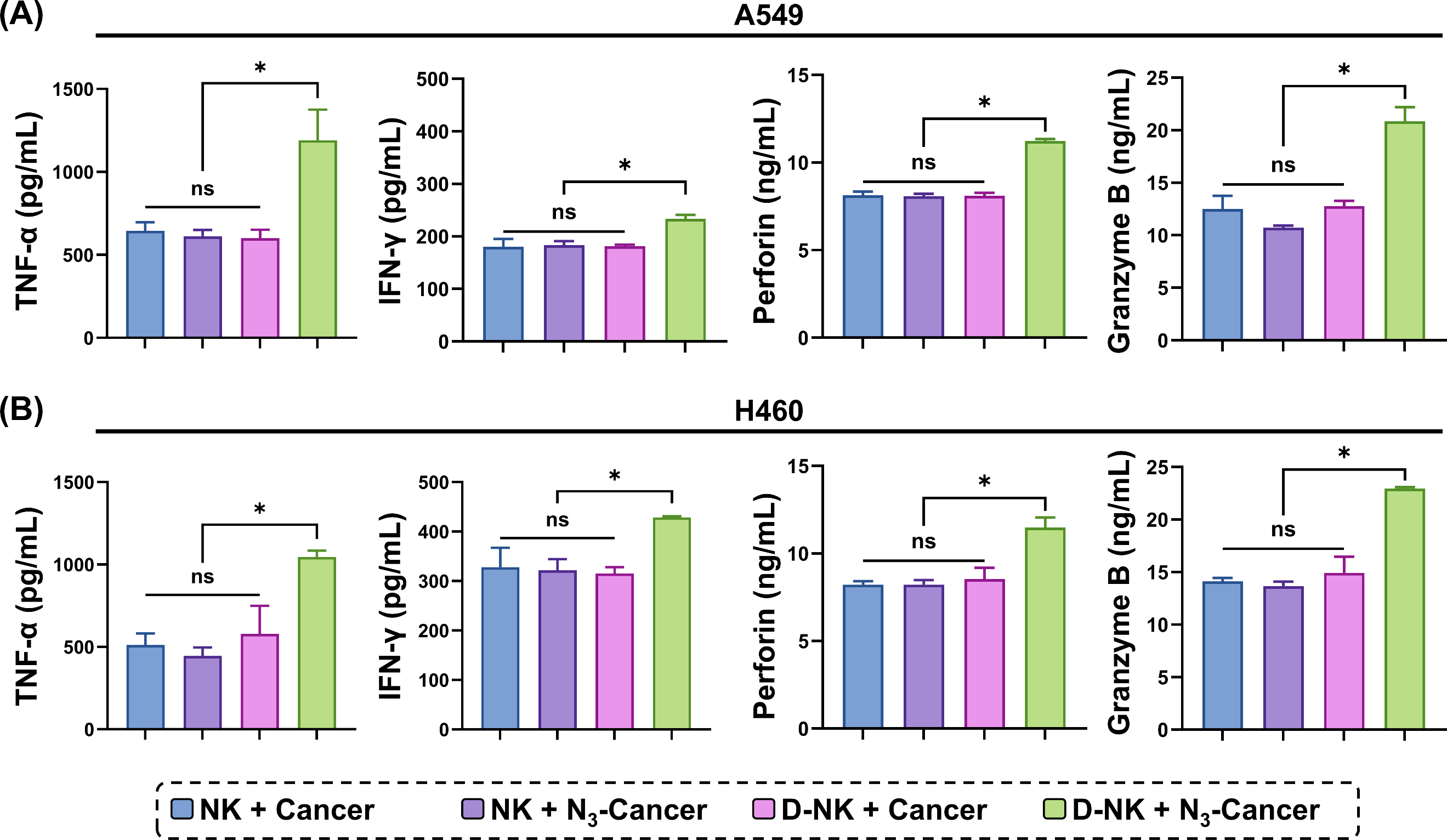


**Fig. S14. Quantification of secreted cytokines and lytic granules from the NK cells after co-culture with (A) A549 or (B) H460 cells, at E:T ratio of 10:1 for 4 h.** The concentrations of secreted cytokines and lytic granules were measured by ELISA kit according to manufacturer’s instructions. Statistical significance was determined by one-way ANOVA followed by Tukey’s multiple comparison test. Differences were considered statistically significant at **p* < 0.05. “ns” indicates statistically non-significant.


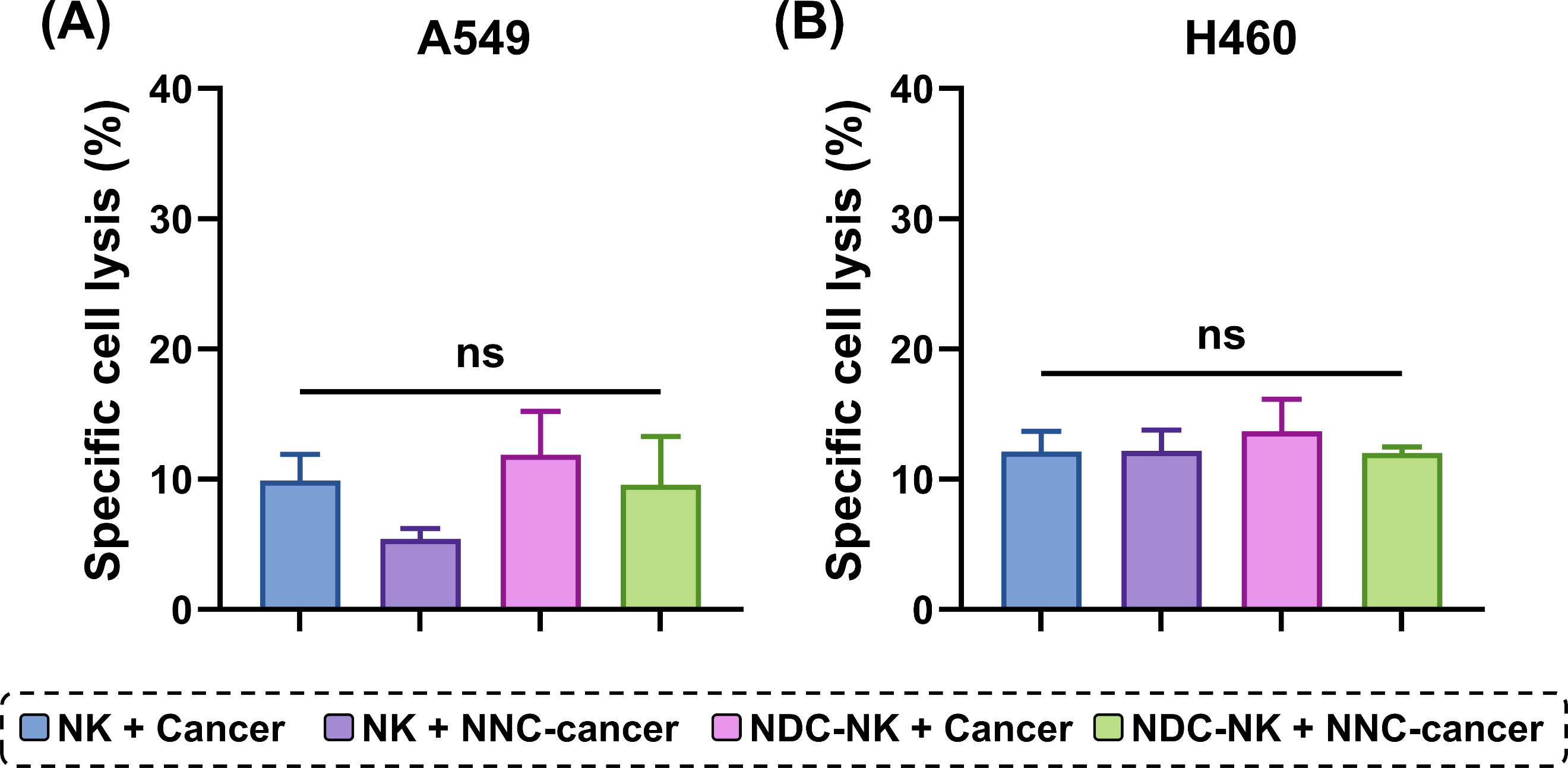


**Fig. S15. In vitro anticancer efficacy of non-reactive lipid analogs-coated NK or cancer cells.** NK cells or non-DBCO lipid-coated NK cells (NDC-NK) co-cultured with unmodified cancer cells or non-N_3_ lipid-coated cancer cells (NNC-cancer) at an E:T ratio of 10:1 for 4 h. NDC-NK cells were prepared using 0.75 mg/mL DSPE-PEG-Gly-Di-PEG-amine intermediate (without the DBCO moiety), and NNC-cancer cells were prepared using 0.75 mg/mL DSPE-PEG-Gly-Di-COOH (without the N_3_ moiety). Specific cell lysis of target (A) A549 and (B) H460 cancer cells by NK and NDC-NK cells is shown. Statistical significance was determined by one-way ANOVA followed by Tukey’s multiple comparison test. Differences were considered statistically significant at **p* < 0.05. “ns” indicates statistically non-significant.


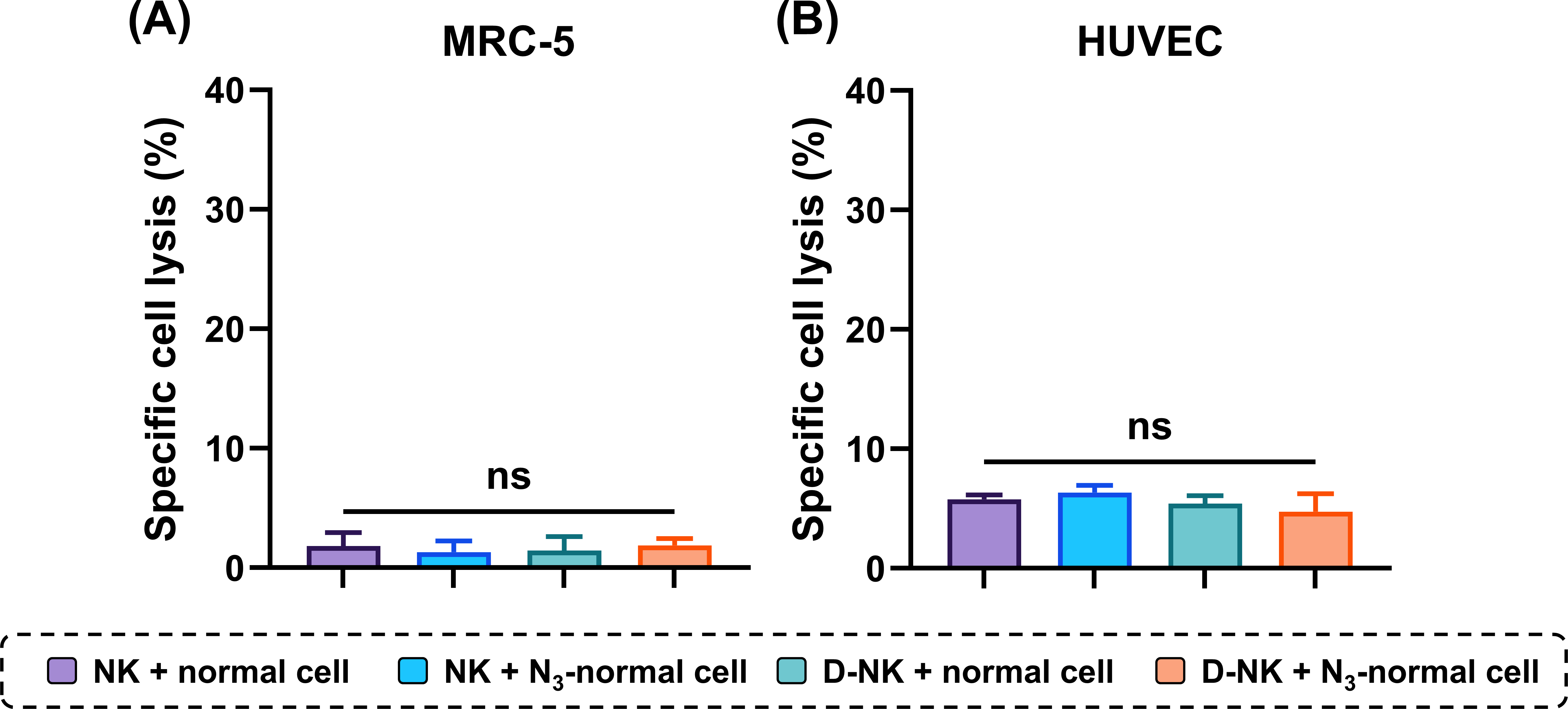


**Fig. S16. Specific cell lysis of normal control cells.** Quantification of (A) normal lung fibroblast (MRC-5) and (B) normal endothelial cell (HUVEC) lysis following 4 h co-culture with NK cells and normal cells at 10:1 E:T ratio. Statistical significance was determined by one-way ANOVA followed by Tukey’s multiple comparison test. Differences were considered statistically significant at **p* < 0.05. “ns” indicates statistically non-significant.


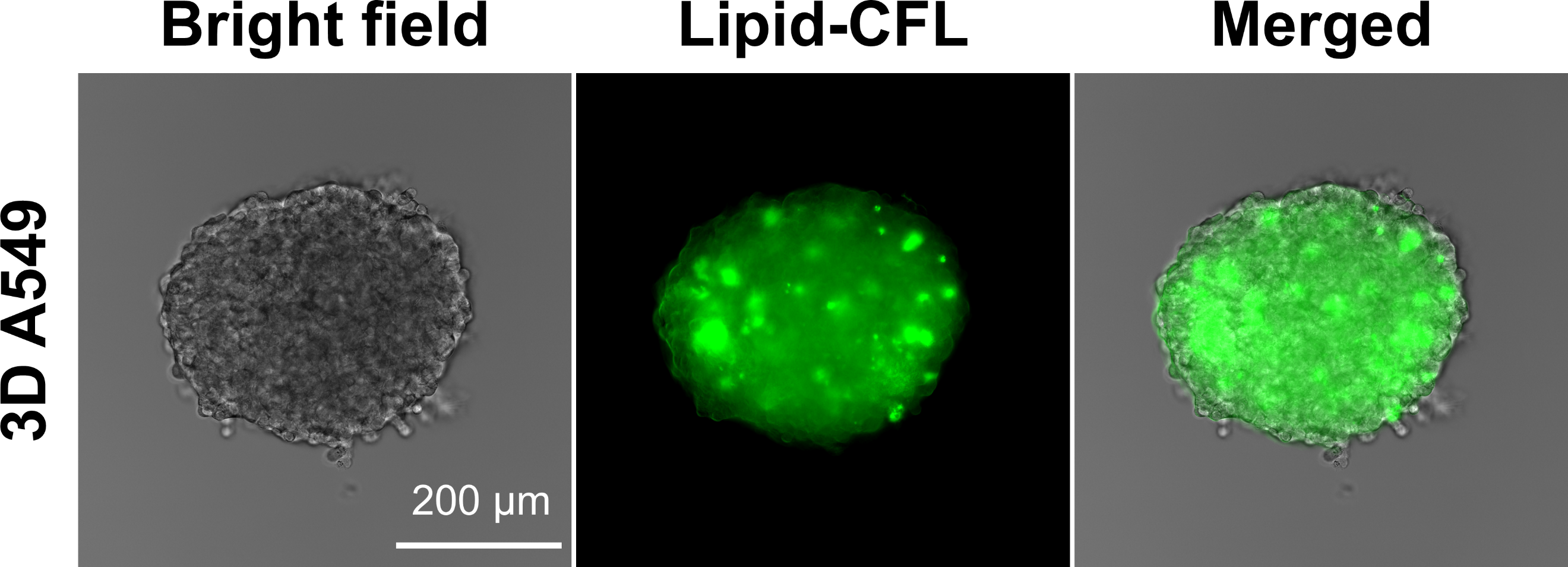


**Fig. S17. 3D A549 lung tumoroid labeled with Lipid-CFL.** Generated 3D lung tumoroids were coated with Lipid-CFL in serum-free medium for 30 min. the fluorescence signals of lung tumoroids surfaces were observed by fluorescence microscope.


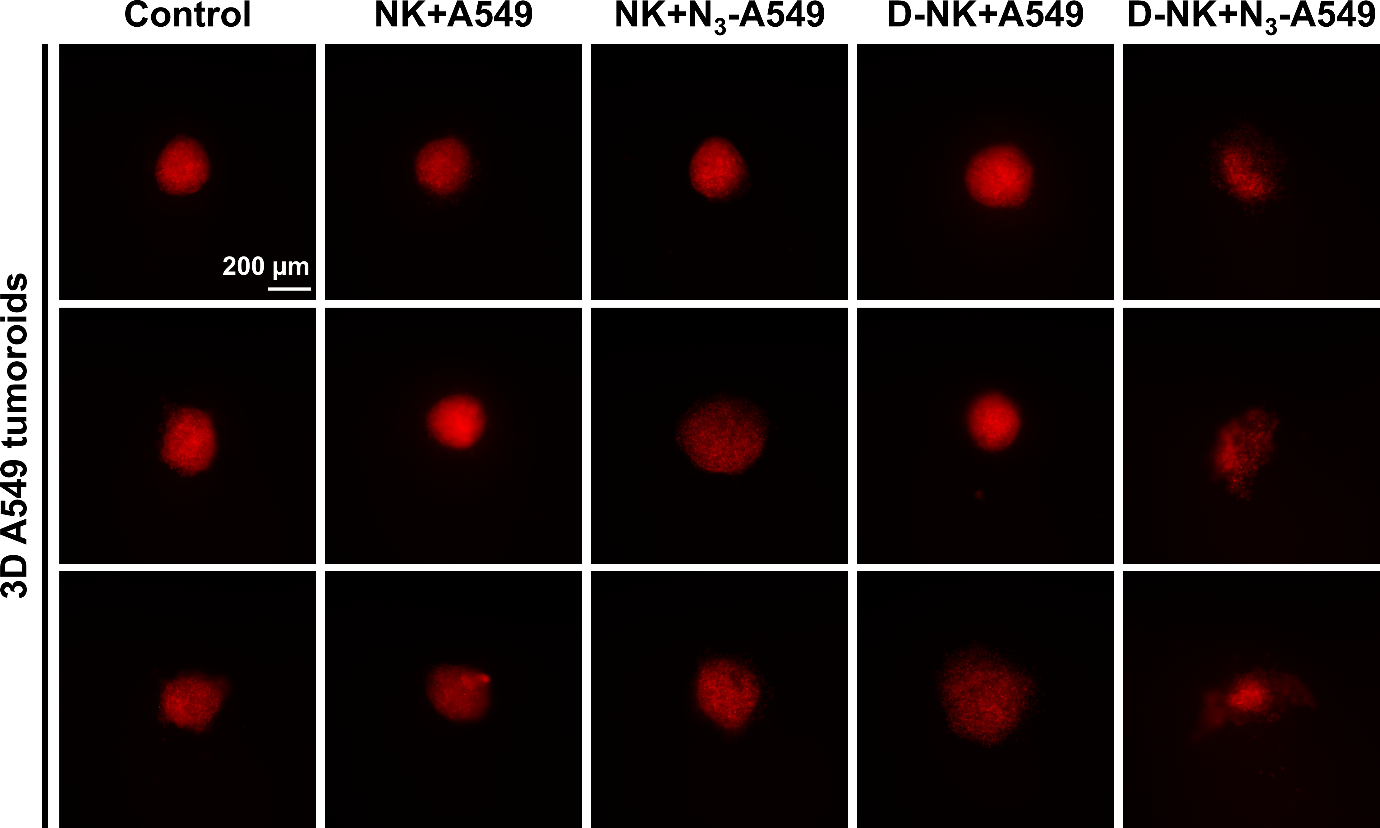


**Fig. S18. Representative fluorescence microscopy images of CellTracker Red-prelabeled 3D A549 tumoroid embedded in collagen-1 gel.** Tumoroid disruption, indicated by reduced CellTracker Red fluorescence intensity, was evaluated following 24 h co-culture with NK or D-NK cells.
